# Molecular Sensory and GC-MS/MS Profiling of Key Aroma Compounds in Sauce-Flavor Base Baijiu

**DOI:** 10.3390/foods15203698

**Published:** 2026-10-17

**Authors:** Na Wang, Yi Shen, Sen Luo, Wei Cheng, Jing Wang, Jian Wang, Zixuan Liu, Hongbo Gao, Bing Liu, Jiao Niu

**Affiliations:** 1 China National Research Institute of Food and Fermentation Industries Co., Ltd., Beijing 100015, China; 15510119560@163.com (N.W.); 2023060585@nwafu.edu.cn (S.L.); wjing2188@163.com (J.W.); onlykissjohn@hotmail.com (J.W.); liuzixuan_97@163.com (Z.L.); 15011559852@139.com (H.G.); 2 College of Food Science and Engineering, Tianjin University of Science and Technology, Tianjin 300457, China; 3 Langjiu Distillery Co., Ltd., Luzhou 646500, China; shenyi@langjiu.cn (Y.S.); 19382756720@163.com (W.C.); 4 College of Food Science and Engineering, Northwest A&F University, Yangling 712100, China

**Keywords:** Sauce-flavor baijiu, GC-MS/MS, key aroma compounds, flavoromics, molecular sensory science

## Abstract

This study systematically investigated the key aroma compounds across seven consecutive production rounds of Sauce-flavor base baijiu from the Tianfu region using an integrated molecular sensory approach. GC-MS profiling tentatively identified 232 volatile compounds by NIST library matching, while 58 low-content and 12 high-content aroma compounds were quantitatively determined by GC-MS/MS and GC-FID, respectively. Thirty-seven compounds showed literature-based OAV values ≥ 1 and were selected as candidate aroma-active compounds for further sensory evaluation. Aroma recombination and omission experiments provided further sensory evidence for the relevance of 13 aroma compounds, including ethyl furfuryl ether, 1-propanol, ethyl isovalerate, and acetoin, to the aroma profiles of representative production rounds. Quantitative analysis revealed marked round-dependent variations in higher alcohols, esters, and acids, highlighting dynamic changes in the volatile composition of Sauce-flavor base baijiu across successive production rounds. This work provides a quantitative and sensory basis for understanding aroma variation across production rounds within the investigated Sauce-flavor baijiu production system and may provide a reference for subsequent flavor evaluation and process optimization.

## 1. Introduction

Sauce-flavor baijiu is a traditional Chinese distilled spirit. It has the production characteristics of “12987” and ultimately yields base baijiu from seven successive production rounds, which is one of the factors that differentiate it from other flavor types [1,2,3]. Distinct variations in flavor profiles have been observed among production rounds. Finished Sauce-flavor baijiu is produced by blending base baijiu collected from different production rounds in appropriate proportions [4]. Consequently, analysis of the key aroma compounds across these rounds provides a basis for controlling and optimizing the flavor properties of Sauce-flavor baijiu.

More than 2700 volatile components have been reported in baijiu [5,6]. However, only a limited subset of key aroma compounds exerts a pronounced influence on baijiu aroma [7]. Molecular sensory science is a cross-disciplinary field dedicated to the investigation of key food flavors at the molecular level. It commonly employs gas chromatography coupled with olfactometry and mass spectrometry (GC-O-MS), together with odor activity values (OAVs) and aroma reconstitution and omission experiments. These approaches are used to elucidate and evaluate the contribution of individual compounds to the overall aroma profile of a sample [8,9,10]. Previous studies have demonstrated substantial differences in sensory characteristics and volatile composition among production rounds of Sauce-flavor baijiu. Liu et al. combined volatile profiling, OAV analysis, multivariate statistics, sensory evaluation, and aroma recombination to characterize aroma differences among production rounds [4]. Gong et al. further combined quantitative analysis, OAV evaluation, and aroma recombination to investigate three typical base baijiu styles [11]. More recently, Li et al. systematically investigated seven production rounds using sensory analysis, aroma extract dilution analysis (AEDA), OAV analysis, and machine learning, identifying 18 key odor-active compounds and establishing models for sensory quality prediction [12]. These studies have substantially advanced the characterization and screening of aroma compounds in Sauce-flavor baijiu. However, experimental verification of the contribution of individual aroma compounds by omission analysis across representative production stages remains limited. Moreover, comprehensive quantitative tracking of aroma compounds across seven consecutive production rounds, followed by sensory verification of selected compounds within the same analytical framework, requires further investigation.

In studies of critical aroma constituents in baijiu, accurate quantification of target constituents was essential for evaluating their aroma contributions and establishing the flavor profile. Nevertheless, conventional GC-MS quantification performed in full-scan or selected ion monitoring (SIM) modes was susceptible to interference from the complex baijiu matrix, which often led to inaccurate quantification results [13]. In selected reaction monitoring (SRM) mode, GC-MS/MS selectively monitors predefined precursor-to-product ion transitions after collision-induced fragmentation, thereby improving selectivity and reducing matrix interference in complex samples [14,15]. Researchers developed a GC-MS/MS approach to quantify 20 pyrazine compounds in baijiu, and the results demonstrated that this approach provided lower detection limits and improved quantitative accuracy, making it more suitable for analyzing flavor compounds in baijiu [16]. Thus, the GC-MS/MS-SRM approach could be used to quantify the compounds more accurately and provide strong technical support for the accurate determination of key aroma compounds and their flavor contributions.

Building on previous studies based on AEDA, OAV analysis, and aroma recombination, the present study focused on integrating quantitative tracking across seven consecutive production rounds with experimental sensory validation of aroma contributors. Specifically, the objectives were to (1) profile volatile compounds in base baijiu from seven production rounds using direct injection and HS-SPME-Arrow-GC-MS; (2) characterize odor-active compounds in representative early, middle, and late production rounds using AEDA and GC-O-MS; (3) quantitatively determine aroma compounds using GC-FID and GC-MS/MS and evaluate their sensory relevance using OAVs; (4) verify the sensory contributions of selected aroma compounds through aroma recombination and omission experiments; and (5) examine associations between the validated compounds and major sensory attributes. This integrated strategy was designed to connect changes in aroma compound concentrations across production rounds with direct sensory evidence from recombination and omission experiments, thereby providing a more experimentally supported characterization of aroma development in Sauce-flavor baijiu.

## 2. Materials and Methods

### 2.1. Preparation of Baijiu Samples

Base baijiu samples from the first to the seventh production rounds of Sauce-flavor baijiu were collected from Langjiu Distillery Co., Ltd., located in Tianfu Village, Maoxi Town, Sichuan, China. According to the conventional Sauce-flavor baijiu production process, the production sequence comprises two initial raw-material feeding stages, nine steaming operations, eight fermentation cycles, and seven successive liquor collection rounds. The first base–baijiu fraction is obtained following the two initial feeding-associated fermentation stages. Thereafter, the fermented grains proceed through repeated fermentation–distillation cycles to generate the second through seventh base–baijiu fractions sequentially, without further raw-material feeding [17,18]. Accordingly, samples obtained from production rounds 1–7 were designated TF-1–TF-7, respectively. The seven samples represented successive stages within one production sequence and were not independent production replicates. The alcoholic strengths of TF-1–TF-7 were 56.6%, 54.5%, 54.5%, 53.5%, 53.9%, 52.5%, and 52.5% (*v*/*v*), respectively. Sampling was conducted in 2024, with 2000 mL collected for each sample, and all samples were kept at −4 °C prior to analysis.

### 2.2. Chemical Reagents and Materials

Anhydrous ethanol, dichloromethane (chromatographic grade, ≥99%) and all analytical and internal standards (chromatographic grade, ≥95% purity) used in the experiments were acquired from Shanghai Aladdin Bio-Chem Technology Co., Ltd. (Shanghai, China). Analytical-grade sodium chloride (NaCl), anhydrous sodium sulfate (Na_2_SO_4_), sodium carbonate (Na_2_CO_3_), and hydrochloric acid (HCl) were purchased from China National Pharmaceutical Group Co., Ltd. (Beijing, China). Sigma-Aldrich Co., Ltd. (Shanghai, China) supplied the C7–C40 n-alkane mixtures used in this study.

### 2.3. Sensory Analysis

The sensory panel consisted of ten trained assessors from the China National Research Institute of Food and Fermentation Industries Co., Ltd. (Beijing, China), aged 23–30 years, and was established in accordance with the GB/T 33404-2016 standard [19]. All ten assessors met the requirements for odor discrimination, intensity discrimination, and descriptive ability.

Sensory evaluation was conducted following previously reported methods with certain modifications [20]. The sensory evaluation was conducted in the sensory analysis laboratory, where the room temperature was maintained at 25 °C. For each sample, each assessor was provided with 30 mL of base baijiu in an individually coded tasting cup and performed the sensory evaluation independently under controlled conditions. Each aroma attribute was evaluated by the panelists using a six-point intensity scale ranging from 0 to 5, where 0 represented the absence of aroma and 5 denoted a very strong intensity, with intermediate scores corresponding to very weak (1), weak (2), moderate (3), and strong (4) perceptions, with higher values corresponding to greater aroma intensity.

Twelve aroma attributes were selected, including sauce aroma, raw grain aroma, ripe grain aroma, raw qu aroma, aged qu aroma, floral aroma, fruity aroma, sweet aroma, sour aroma, green aroma, Zao aroma, and baked aroma.

### 2.4. Analysis of Volatile Compounds by GC-MS

#### 2.4.1. Sample Preparation

For direct injection, each baijiu sample was fortified with 2-propylpyrazine (IS1) and 3,4-dimethylphenol (IS2), each at a concentration of 1 mg/L, prior to GC-MS analysis.

The HS-SPME-Arrow pretreatment was performed according to Zhang et al. [21], with minor modifications. Each sample was diluted to an ethanol content of 10% (*v*/*v*) with ultrapure water. A 5 mL aliquot of the diluted solution was combined with 1.50 g of NaCl in a standard 20 mL headspace sampling vial, then spiked with 2-propylpyrazine (IS1) and 3,4-dimethylphenol (IS2) at a concentration of 1 mg/L as internal standard compounds. After equilibration by shaking at 45 °C for 5 min, the mixture underwent solid-phase microextraction (SPME) using a 1.10 mm DVB/CWR/PDMS fiber at 45 °C for 45 min. The fiber was then desorbed at 250 °C for 5 min, and the extracted components were subsequently separated and detected by GC-MS.

#### 2.4.2. GC-MS Analysis

GC-MS analysis was performed on a TRACE 1600-TSQ 9610 gas chromatograph coupled with a triple-quadrupole mass spectrometer and a TriPlus™ RSH autosampler (Thermo Fisher Scientific, Waltham, MA, USA). Samples were separated using a TG-WAXMS capillary column (60 m × 0.25 mm × 0.25 μm; Thermo Fisher Scientific, USA). A 1 µL portion of each sample was injected in splitless mode at 250 °C, with helium (99.999%) flowing at 1.0 mL/min as the carrier gas. The oven temperature program started at 35 °C for 1 min, increased to 150 °C at 2 °C/min (held for 10 min), and then to 230 °C at 5 °C/min (held for 15 min), for a total duration of 99.5 min. MS detection was performed under electron ionization at 70 eV, with the transmission line set at 240 °C and ion source at 250 °C, scanning from m/z 30 to 300 amu.

Volatile compounds were tentatively identified by comparing their EI mass spectra with the NIST 2020 mass spectral library. Semiquantitative analysis was performed using the internal-standard method. 2-Propylpyrazine (IS1) and 3,4-dimethylphenol (IS2), each at a final concentration of 1 mg/L, were used as internal standards. Compounds with retention times < 45 min were semiquantified using IS1, whereas compounds with retention times ≥ 45 min were semiquantified using IS2. The semiquantitative concentration of each compound was calculated from the ratio of its peak area to that of the corresponding internal standard, assuming a relative response factor of 1. The resulting values were considered semiquantitative internal-standard equivalents rather than compound-specific validated concentrations.

### 2.5. AEDA-GC-O-MS Analysis

#### 2.5.1. LLE Experiment

Aroma compounds from base baijiu of different production rounds were enriched using a modified LLE procedure, originally described by Chen et al. [22]. Specifically, a 50 mL aliquot of each selected base baijiu sample (TF-1, TF-4, and TF-7) was diluted to 15% (*v*/*v*) with ultrapure water, and saturated NaCl was added. The solution was extracted three times with CH_2_Cl_2_, and the combined organic layers were washed sequentially three times with 0.50 mol/L Na_2_CO_3_ (pH 10.0) and once with 35 mL of saturated NaCl to obtain the neutral/basic fraction (NBF). The remaining aqueous phase was acidified to pH 2.0 with 4.0 mol/L HCl and extracted three times with CH_2_Cl_2_, and the collected extracts were pooled as the acidic fraction (AF). After drying over anhydrous Na_2_SO_4_, both the neutral/basic and acidic fractions were stored overnight at −4 °C. The volumes were reduced to 1.0 mL with a rotary evaporator (BÜCHI Labortechnik AG, Flawil, Switzerland).

#### 2.5.2. GC-O-MS Analysis

GC-O-MS experiments were performed on a QP 2010 GC-MS system (Shimadzu Co., Ltd., Kyoto, Japan) coupled with an OP 275 Pro II olfactometry instrument (Guangzhou Ingenious Laboratory Technology Co., Ltd., Guangzhou, China). A TG-WAXMS capillary column (60 m × 0.25 mm i.d., 0.25 μm film thickness; Thermo Fisher Scientific, USA) was used for separation. The method was adapted from a previously published study with minor modifications [23]. For GC analysis, 1 µL of sample was injected without splitting at 250 °C, with helium (99.999%) as the carrier gas at 1.0 mL/min. The oven program started at 50 °C (1 min), ramped to 125 °C at 10 °C/min (held 4 min), then to 200 °C at 3 °C/min, and finally to 230 °C at 15 °C/min (10 min hold). Mass spectrometry conditions were as described in Section 2.4.2.

In olfactometry, the sniffing port was maintained at 250 °C, and humidified air was supplied to the sniffing port at 50 mL/min. The effluent was split equally (1:1) between the detector and sniffing port. Aroma identification combined MS spectra from the NIST 2020 library, retention indices, odor notes, and comparisons with authentic standards.

#### 2.5.3. AEDA Analysis

The NBF and AF extracts were serially diluted threefold with CH_2_Cl_2_ (FD = 1 for the undiluted extract, followed by 3, 9, 27, 81, 243, …) until the odor event no longer met the reproducibility criterion. Two experienced assessors conducted the GC-O-MS analysis independently. Each assessor evaluated every dilution twice, resulting in four evaluations for each dilution level. An odor event was considered reproducibly detected when it was perceived in at least three of the four evaluations. The FD factor was defined as the highest dilution factor at which this reproducibility criterion was satisfied. When the assessors initially provided different odor descriptors for the same odor event, the corresponding chromatographic region was re-evaluated until a consensus descriptor was obtained. For each odor event, the retention time and consensus odor descriptor were recorded. FD factors were used as relative indicators of odor persistence in the AEDA extracts and were not interpreted as direct quantitative measures of sensory contribution in the original baijiu matrix [24].

### 2.6. Quantitative Analysis of Aroma Compounds

#### 2.6.1. GC-FID Analysis

GC-FID analyses were carried out on an A 91 Plus gas chromatograph equipped with a flame ionization detector (Panna (Changzhou) Instruments Co., Ltd., Changzhou, China). A DB-WAX capillary column (60 m × 0.25 mm × 0.25 μm; Agilent Technologies, Inc., Santa Clara, CA, USA) was used for separation. Baijiu samples were spiked with a mixture of tertiary amyl alcohol (IS3), amyl acetate (IS4), and 2-ethylbutanoic acid (IS5) to a final concentration of 200 mg/L. A 1 μL portion of the spiked sample was injected in split mode (split ratio 20:1). Nitrogen (99.999%) was used as the carrier gas at 1.0 mL/min, with hydrogen at 30 mL/min and air at 300 mL/min. The oven was programmed from 35 °C (1 min) to 230 °C at 3.5 °C/min, held for 10 min, while the injector and detector were maintained at 250 °C. Whenever the response of an individual analyte exceeded its upper calibration limit, a separate aliquot of the sample was diluted 50-fold and reanalyzed for that analyte. Analytes whose responses were already within their respective calibration ranges were quantified from the corresponding undiluted preparation. For the diluted aliquot, the internal-standard mixture was added after dilution to achieve the specified final internal-standard concentration in the solution subjected to GC-FID analysis.

#### 2.6.2. GC-MS/MS Analysis

GC-MS/MS quantification was performed on a TRACE 1600 gas chromatograph coupled to a TSQ 9610 triple quadrupole mass spectrometer (Thermo Fisher Scientific, USA), using a TG-WAXMS capillary column (60 m × 0.25 mm × 0.25 μm; Thermo Fisher Scientific, USA). Samples requiring dilution were first diluted by the appropriate factor. 2-Propylpyrazine (IS1) and 3,4-dimethylphenol (IS2) were subsequently added so that each internal standard was present at a final concentration of 1 mg/L in the solution subjected to GC-MS/MS analysis. Calibration standards and unknown samples were prepared and calculated using the same internal-standard procedure. Gas chromatography conditions were applied as described in Section 2.5.2. Mass spectrometric detection was conducted under electron ionization at 70 eV, with the transmission line and ion source temperatures set at 240 °C and 260 °C, respectively. Data were collected in SRM mode, with detailed acquisition parameters provided in Table 1. For analytes exceeding the upper calibration limit, the samples were diluted tenfold before GC-MS/MS analysis; propyl lactate was diluted twentyfold because of its higher concentration. The concentrations in the analyzed solutions were maintained within the validated calibration range, and the concentrations in the original samples were calculated using the corresponding dilution factors. No quantitative values were obtained by extrapolation beyond the calibration range.

#### 2.6.3. Method Validation

The quantitative methods were validated for linearity, recovery, and precision. Limits of detection (LODs) and quantification (LOQs) were additionally evaluated for the GC-MS/MS method. LODs and LOQs were not determined for the GC-FID method because GC-FID was applied specifically to high-content aroma compounds, for which trace-level sensitivity was not an analytical objective. Calibration curves for GC-FID were constructed using at least 10 concentration levels spanning the validated quantitative range of each analyte, from the lower to the upper calibration limit, with intermediate concentrations distributed across the corresponding range. The validated calibration ranges and regression equations are provided in Table S1. For GC-MS/MS, nine calibration levels of 0.003, 0.01, 0.03, 0.10, 0.30, 1.0, 3.0, 6.0, and 9.0 mg/L were used (Table S2).

For the GC-MS/MS method, the limits of detection (LOD) and quantification (LOQ) were evaluated using a 10 mg/L mixed standard solution and were determined on the basis of signal-to-noise ratios of 3 and 10, respectively.

A pooled quality-control (QC) sample was prepared by mixing equal volumes of the seven Sauce-flavor base baijiu samples (TF-1–TF-7). Method precision was evaluated using the pooled QC sample. Intraday precision was determined from five independent replicate measurements performed within the same day, whereas interday precision was evaluated by analyzing the QC sample on five consecutive days. Precision was expressed as the relative standard deviation (RSD).

Recovery was evaluated by spiking the pooled QC sample at a representative mid-range calibration level for each target analyte. The spike concentrations were 1000 mg/L for ethyl acetate; 100 mg/L for 2-butanol and furfural; 3000 mg/L for 1-propanol; 100 mg/L for 2-methyl-1-propanol and ethyl palmitate; 40 mg/L for 1-butanol, propanoic acid, and 2,3-butanediol; 200 mg/L for 3-methyl-1-butanol; 800 mg/L for ethyl lactate; 1000 mg/L for acetic acid; and 0.30 mg/L for the compounds quantified by GC-MS/MS. Recovery was calculated as the percentage of the measured increase in analyte concentration relative to the added concentration.

### 2.7. Calculation of OAVs

OAVs were calculated as the ratio between the concentration of an aroma compound and its odor threshold. The odor thresholds used in this study were obtained from the previously published literature rather than determined directly in the investigated Sauce-flavor baijiu matrices [5]. Therefore, OAVs were used as screening indices to prioritize compounds for further sensory evaluation. Compounds with OAV ≥ 1 were considered candidate aroma-active compounds, but the absolute OAV values were not interpreted as direct quantitative measures of sensory contribution because odor thresholds may vary with matrix composition, ethanol concentration, and sensory evaluation conditions.

### 2.8. Aroma Recombination and Omission Tests

Aroma recombination models were prepared for TF-1, TF-4, and TF-7 using hydroalcoholic matrices containing 56.6%, 53.5%, and 52.5% ethanol (*v*/*v*), respectively, corresponding to the alcoholic strengths of the original samples. Compounds with OAV ≥ 1 and quantitatively determined concentrations were included in each model and added at the concentrations measured in the corresponding original sample. Accordingly, the TF-1, TF-4, and TF-7 recombination models contained 34, 35, and 37 aroma compounds, respectively. Compounds without a reliable quantitative concentration above the validated quantification limit were not assigned concentrations by extrapolation and were not used for model preparation. The identities and final concentrations of all compounds included in the recombination models are provided in Table S6. The aroma profiles of the original samples and the corresponding recombination models were evaluated by the sensory panel using the 0–5 intensity scale described in Section 2.3. The hydroalcoholic matrices reproduced the ethanol concentrations of the original samples but did not reproduce the complete nonvolatile matrix composition of the original Sauce-flavor baijiu. Therefore, the recombination mixtures were considered simplified aroma models under matched ethanol conditions.

For the omission experiments, one aroma compound was removed at a time from the corresponding complete recombination model, while the concentrations of all remaining compounds and the ethanol concentration of the matrix were kept unchanged. A triangle test was then performed using two complete recombination samples and one omission sample [25]. Ten assessors were asked to identify the sample that differed from the other two. Statistical significance was evaluated using the exact binomial probability with a chance probability of 1/3. For ten assessors, 7, 8, and ≥9 correct responses were considered significant at *p* < 0.05, *p* < 0.01, and *p* < 0.001, respectively.

### 2.9. Statistical Analysis

For the instrumental analyses, three independent analytical preparations were made from each base baijiu sample and analyzed separately. These triplicates represented analytical replication of the same sample and were not independent production replicates. Quantitative results are expressed as the mean ± standard deviation of the three analytical replicates. The seven production rounds represented successive stages of one production sequence.

For the sensory evaluation data, because the same 10 trained assessors evaluated all seven production-round samples using an ordinal six-point intensity scale (0–5), differences among production rounds were analyzed separately for each sensory attribute using the Friedman test. Kendall’s W was calculated as the effect-size measure for the overall production-round effect. The *p*-values obtained from the 12 Friedman tests were adjusted using the Holm procedure to account for multiple testing. Pairwise comparisons among the seven production rounds were performed using paired Wilcoxon signed-rank tests, with Holm adjustment applied to the 21 pairwise comparisons within each sensory attribute. Zero paired differences were excluded, and tied absolute differences were assigned average ranks. Exact two-sided significance for the pairwise signed-rank tests was evaluated by exhaustive sign permutation of the non-zero paired differences. Adjusted *p* < 0.05 was considered statistically significant. Pairwise differences are presented using lowercase grouping letters, with production rounds sharing at least one letter considered not significantly different. The raw assessor-level sensory intensity scores used for these statistical analyses are provided in Table S7.

**Figure 1:**
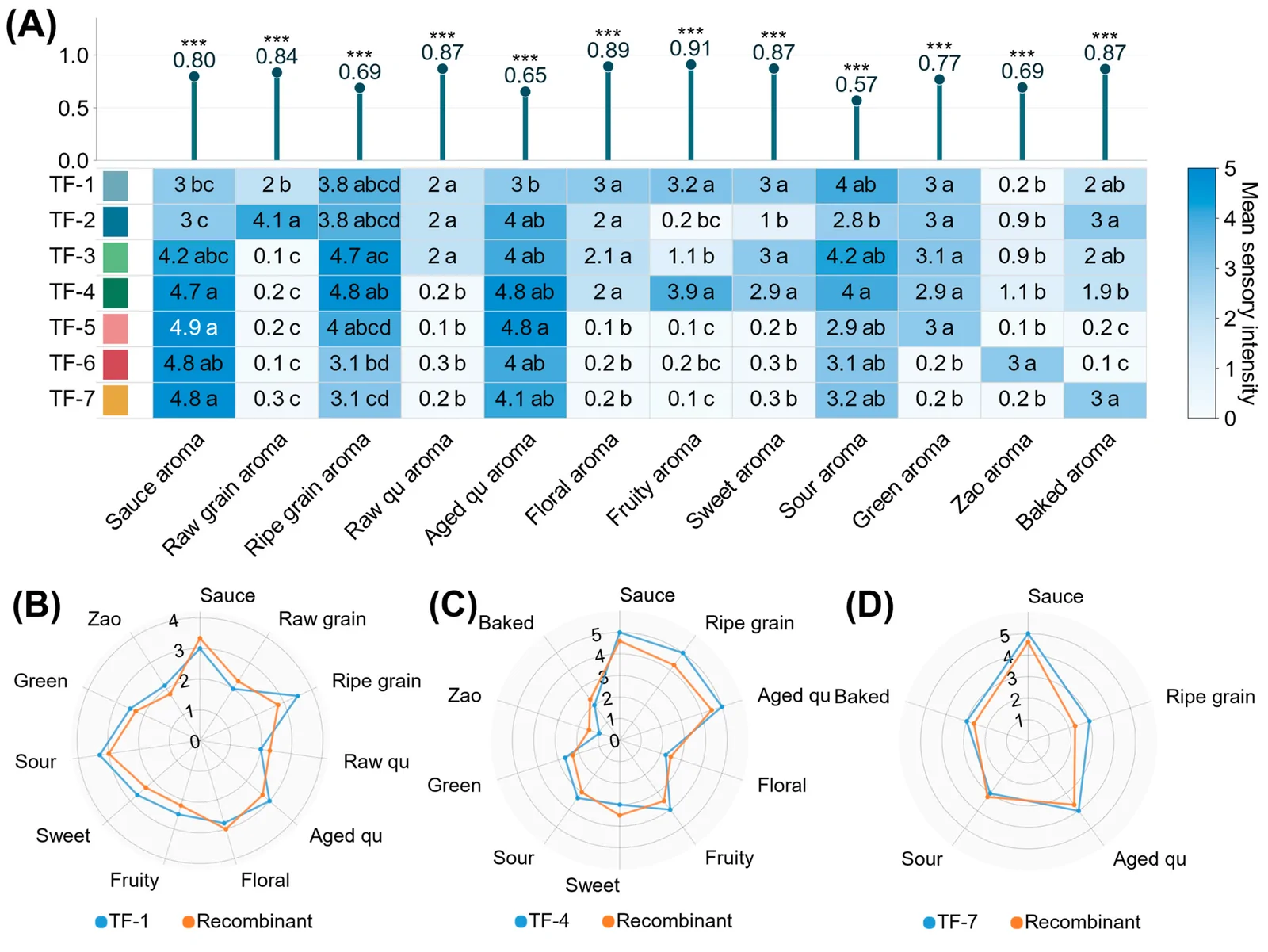
Sensory profiles of Sauce-flavor base baijiu across production rounds and corresponding aroma recombination models. (**A**) Sensory profiles and statistical differences among TF-1–TF-7 determined by quantitative descriptive analysis. Cell values represent mean sensory intensities; lowercase letters indicate pairwise statistical groupings within each sensory attribute, and the upper panel shows Kendall’s W and Holm-adjusted significance of the overall production-round effect; (**B**–**D**) comparison of the original samples and aroma recombination models for TF-1, TF-4, and TF-7, respectively. *** indicates Holm-adjusted *p* < 0.001.

Sensory statistical analyses were performed in Python 3.12.7 (Python Software Foundation, Wilmington, DE, USA) using NumPy 2.1.3, pandas 2.2.3, SciPy 1.14.1, and statsmodels 0.14.4. Friedman tests were conducted using scipy.stats.friedmanchisquare, tied absolute differences for pairwise signed-rank analysis were ranked using scipy.stats.rankdata, and Holm multiple-comparison adjustment was performed using statsmodels.stats.multitest.multipletests with method = “holm”. Kendall’s W was calculated as W = χ^2^/[*n* × (k − 1)], where χ^2^ is the Friedman test statistic, *n* is the number of assessors, and k is the number of production rounds. Pairwise grouping letters were generated from the Holm-adjusted pairwise comparison results using a custom Python routine. Figure 1A was generated using Matplotlib 3.9.2.

For Spearman correlation analysis, the analytical replicates were averaged within each production round for each aroma compound. For each sensory attribute, the scores obtained from the ten assessors were averaged within each production round. The seven production rounds were then treated as the effective observations (*n* = 7). Spearman’s rank correlation coefficients (ρ) were calculated between the 13 key aroma compounds and 12 sensory attributes, yielding 156 compound–attribute associations. Given the limited effective sample size and the large number of associations examined, the correlation analysis was treated as exploratory and the coefficients were interpreted descriptively in terms of the direction and magnitude of co-variation rather than as evidence of causal or sensory-interaction effects. For multiple-testing assessment, the corresponding *p* values were additionally adjusted using the Benjamini–Hochberg procedure [26]. Spearman correlations were calculated in Python 3.12.7 using scipy.stats.spearmanr. For visualization, the rows and columns of the correlation matrix were independently ordered by average-linkage hierarchical clustering using Euclidean distances between their correlation profiles.

The X matrix used for OPLS-DA consisted of the semiquantitative volatile profile data obtained by GC-MS using the internal-standard response-ratio method described in Section 2.4.2. Before OPLS-DA, the X-variable data were log-transformed, mean-centered, and scaled to unit variance (UV). Model fitting was performed in SIMCA 14.1 using the software’s default sevenfold cross-validation scheme, in which every seventh observation was assigned to the same cross-validation group. The number of model components was selected using the Autofit procedure according to the built-in cross-validation criterion. The default 95% confidence level and significance level of 0.05 were retained. Model robustness was additionally examined using 200 Y-permutation tests. OPLS-DA was subsequently performed using SIMCA 14.1 (Umetrics, Umeå, Sweden) as an exploratory multivariate analysis to visualize differences in volatile profiles among production stages and the repeatability of the analytical measurements. Other heatmaps for instrumental and multivariate data visualization were generated using Origin 2024 (OriginLab Co., Ltd., Northampton, MA, USA).

## 3. Results and Discussion

### 3.1. Sensory Profiles of Base Baijiu Across Production Rounds Determined by Quantitative Descriptive Analysis (QDA)

Quantitative descriptive analysis was performed to characterize the sensory profiles of base baijiu across the seven production rounds (Figure 1). The individual assessor-level sensory intensity scores underlying these analyses are provided in Table S7. The Friedman tests revealed significant overall production-round effects for all 12 sensory attributes after Holm adjustment (all adjusted *p* < 0.001). Kendall’s W ranged from 0.57 to 0.91, indicating attribute-dependent differences in the magnitude of the production-round effect. The effect sizes were 0.80 for sauce aroma, 0.84 for raw grain aroma, 0.69 for ripe grain aroma, 0.87 for raw qu aroma, 0.65 for aged qu aroma, 0.89 for floral aroma, 0.91 for fruity aroma, 0.87 for sweet aroma, 0.57 for sour aroma, 0.77 for green aroma, 0.69 for Zao aroma, and 0.87 for baked aroma.

Pairwise comparisons further characterized the sensory transitions among production rounds. Sauce aroma increased from mean intensities of 3.0 in TF-1 and TF-2 to 4.7–4.9 in TF-4–TF-7, although overlapping compact-letter groups were observed for TF-3 and TF-6. Raw grain aroma was highest in TF-2 (4.1, group a), intermediate in TF-1 (2.0, group b), and low in TF-3–TF-7 (0.1–0.3, group c). Raw qu aroma showed a clear early-round pattern, with TF-1–TF-3 assigned to group a and TF-4–TF-7 to group b. Floral aroma was also higher during TF-1–TF-4 than during TF-5–TF-7.

Several attributes showed more complex or non-monotonic patterns. Fruity aroma was highest in TF-1 and TF-4, whereas sweet aroma was higher in TF-1, TF-3, and TF-4 than in TF-2 and TF-5–TF-7. Green aroma separated TF-1–TF-5 from TF-6–TF-7, while Zao aroma was markedly elevated in TF-6. Baked aroma was highest in TF-2 and TF-7 and lowest in TF-5 and TF-6. Although sour aroma showed a significant overall Friedman effect, most production rounds showed overlapping pairwise groups after Holm adjustment, with the clearest separation occurring between TF-2 and TF-4.

Collectively, these statistical results indicate that sensory evolution across the seven production rounds was attribute-specific rather than uniformly monotonic. The early rounds were distinguished by stronger raw grain and raw qu characteristics; the middle rounds showed enhanced sauce, ripe grain, and aged qu aromas; and the later rounds retained strong sauce aroma while several grain-, floral-, fruity-, and green-related attributes weakened.

Previous studies have demonstrated that successive production rounds of Sauce-flavor baijiu are accompanied by substantial changes in microbial community composition, fermentation substrates, and flavor metabolite profiles [17,18,27]. In particular, changes in ester and pyrazine production have been reported during the early and middle rounds, while the later rounds also exhibit distinct microbial communities and metabolite compositions. These fermentation dynamics may contribute to the production-round-dependent sensory patterns observed among production rounds. However, because microbial communities and fermentation metabolites were not directly measured in the present study, these mechanisms are considered literature-supported explanations rather than experimentally demonstrated processes.

### 3.2. Characterization of Volatile Compounds Across Production Rounds by GC-MS

#### 3.2.1. Characterization of Volatile Compounds Across Production Rounds

**Figure 2:**
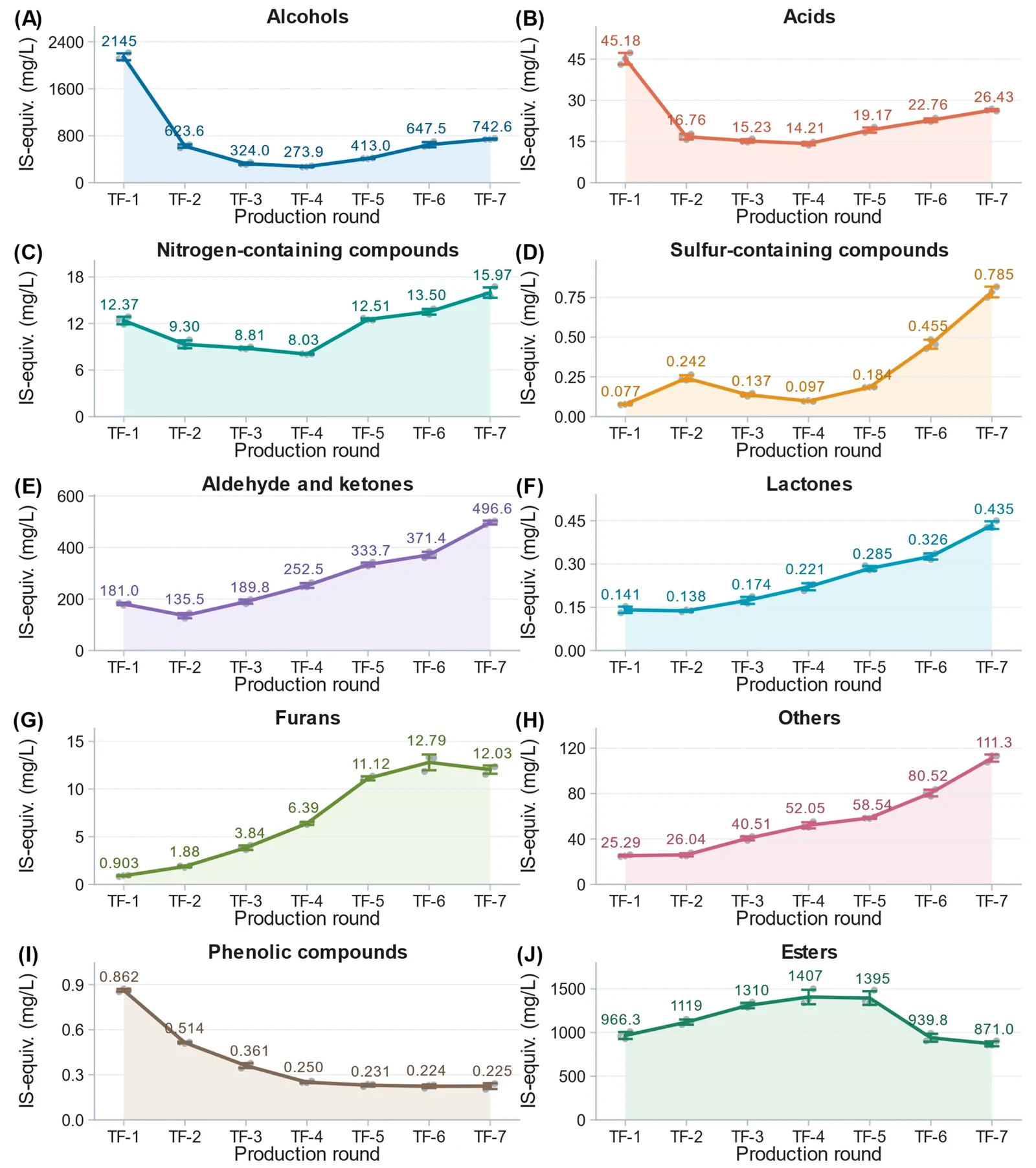
Changes in the semiquantitative levels of major volatile compound classes across seven production rounds of Sauce-flavor base baijiu. (**A**) Alcohols; (**B**) acids; (**C**) nitrogen-containing compounds; (**D**) sulfur-containing compounds; (**E**) aldehydes and ketones; (**F**) lactones; (**G**) furans; (**H**) other compounds; (**I**) phenolic compounds; and (**J**) esters. Lines connect the mean semiquantitative levels across TF-1–TF-7, error bars represent standard deviations of three analytical replicates, and individual points indicate replicate measurements. Values represent semiquantitative internal-standard equivalents calculated from analyte-to-internal-standard peak-area ratios (mg/L, IS-equiv.).

A total of 232 volatile compounds were tentatively identified across the seven production rounds by GC-MS using a combination of direct injection and HS-SPME-Arrow extraction. These compounds comprised 87 esters, 45 alcohols, 21 acids, 38 aldehydes and ketones, 11 nitrogen-containing compounds, 5 furans, 5 phenolic compounds, 2 lactones, 2 sulfur-containing compounds, and 16 compounds from other chemical classes. Among the 232 compounds, 183 were detected in all seven production rounds. The total numbers of volatile compounds detected in TF-1–TF-7 were 212, 210, 221, 229, 223, 223, and 213, respectively, indicating substantial overlap in volatile composition together with variation among production rounds. Such round-dependent variation is consistent with previous studies showing that the microbial community, physicochemical environment, and flavor metabolite profiles vary throughout the multiround fermentation process of Sauce-flavor baijiu [17,27,28]. Changes in the semiquantitative levels of the major volatile compound classes across production rounds are presented in Figure 2. These values were obtained using the internal-standard semiquantification procedure described in Section 2.4.2. Therefore, the values shown in Figure 2 represent semiquantitative internal-standard equivalents rather than validated absolute concentrations. Alcohols, acids, and nitrogen-containing compounds decreased from TF-1 to TF-4 and subsequently increased. Sulfur-containing compounds showed a non-monotonic pattern, with an initial increase at TF-2, a decrease to TF-4, and a marked increase during the later production rounds. Aldehydes and ketones, lactones, and other compounds generally increased toward the later rounds and reached their highest levels in TF-7, whereas furans increased to a maximum in TF-6 and decreased slightly in TF-7. Phenolic compounds declined markedly from TF-1 to TF-4 and changed only slightly thereafter. Esters increased progressively to TF-4 and subsequently decreased.

#### 3.2.2. Exploratory OPLS-DA of Volatile Profiles Across Production Rounds

**Figure 3:**
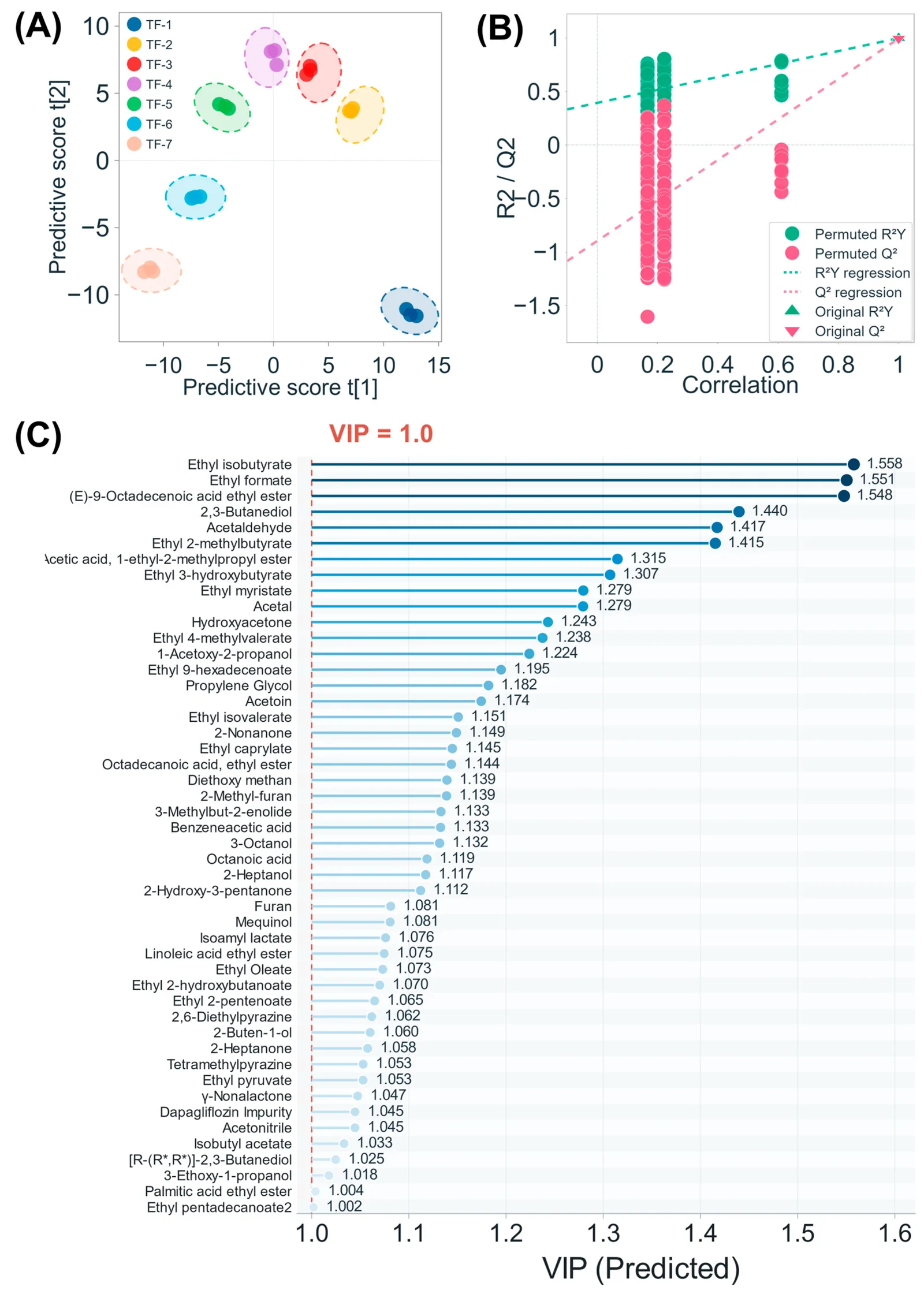
Exploratory OPLS-DA of volatile profiles in Sauce-flavor base baijiu from seven production rounds. (**A**) Score plot based on the first two predictive components; (**B**) results of 200 permutation tests used to assess internal model validity; and (**C**) ranked VIP values of volatile compounds contributing to the observed multivariate separation. Compounds with VIP > 1 are shown in descending order, and the dashed vertical line denotes the VIP = 1 screening threshold. The model contained six predictive components and one orthogonal component in X, with R^2^X(cum) = 0.973, R^2^Y(cum) = 0.981, and Q^2^(cum) = 0.945. The R^2^ and Q^2^ intercepts of the permutation test were 0.394 and −0.895, respectively. A VIP value > 1 was used as the screening criterion for variable contribution. The asterisks () in “[R-(R*,R)]-2,3-butanediol” are part of the stereochemical nomenclature and do not indicate statistical significance.

OPLS-DA was performed to explore differences in volatile profiles among the seven production rounds (Figure 3A). The final model contained six predictive components and one orthogonal component in X, with R^2^X(cum) = 0.973, R^2^Y(cum) = 0.981, and Q^2^(cum) = 0.945. TF-1 showed the greatest multivariate separation from the remaining production rounds, whereas the second to fifth rounds showed relatively smaller differences, and the sixth and seventh rounds were also more uniform, consistent with previous reports [4]. The 200 permutation tests yielded R^2^ and Q^2^ intercepts of 0.394 and −0.895, respectively (Figure 3B). The negative Q^2^ intercept, together with the permutation results, supported the internal validity of the fitted model; however, the permutation test was used to assess internal model performance rather than external predictive validity. A total of 48 compounds showed VIP values > 1 (Figure 3C), indicating relatively high contributions to the multivariate separation observed in the current model. These compounds were, therefore, considered candidate variables associated with the observed differences among the investigated production stages rather than independently validated differential markers. Because the seven production rounds originated from one production sequence and the repeated measurements were analytical replicates, the OPLS-DA results should be interpreted as exploratory patterns within the investigated system. Validation using independent production batches would be required before these variables could be considered generalizable markers of production round.

### 3.3. Characterization of Odor-Active Compounds in Representative Production Rounds by AEDA-GC-O-MS

The distinctive flavor of baijiu is largely determined by its key aroma compounds. In this work, base baijiu from TF-1, TF-4, and TF-7, representing early, middle, and late production rounds, respectively, was selected for further investigation. Their main odor-active compounds were analyzed using the AEDA-GC-O-MS method. A total of 76 odor-active compounds were characterized, including 20 esters, 15 alcohols, 8 acids, 11 nitrogen-containing compounds, 7 aldehydes and ketones, 7 phenolics, 4 furans, 2 lactones, 1 sulfur-containing compound, and 1 compound from another chemical class. Among them, 70 compounds were confirmed using MS, RI, consensus odor descriptors, and authentic standards, whereas 6 compounds were tentatively identified using MS and odor information without authentic standard confirmation. The corresponding FD factors and identification information are presented in Table 2.

A total of 53 odor-active compounds were characterized in the first round of base baijiu samples, including 15 esters, 14 alcohols, 6 acids, 6 nitrogenous compounds, 3 aldehydes and ketones, 5 phenolics, 2 furans, 1 lactone, and 1 compound from another chemical class. Among them, three compounds had FD values of 243, namely ethyl acetate (fruity, with baijiu aroma), 3-methyl-1-butanol (burnt, malty), and γ-nonalactone (coconut aroma). These compounds, therefore, exhibited particularly persistent odor activity in the TF-1 AEDA extract and were retained for subsequent quantitative and sensory evaluation. Thirteen compounds were found exclusively in the first-round base baijiu samples, namely propyl propionate (apple, banana), 4-ethoxy-2-butanone (fruity), isobutyl lactate (butter, caramel), 2,6-diethylpyrazine (baked, soil), 2-methyl-4-octanol (fruity, floral), propanoic acid (sour odor, sweat), diethyl succinate (fruity, slightly sweet), guaiacol (burnt, wood), creosol (fumigate, clove), 2-acetylpyrrole (nutty, brewed aroma), 4-ethyl-2-methoxyphenol (fumigate, roasted), 4-phenyl-3-buten-2-ol (fruity, floral), and 2-methoxy-4-vinylphenol (spice, fried peanuts). These odor-active compounds were detected specifically in TF-1 under the present analytical conditions.

A total of 50 odor-active compounds were detected in TF-4, representing the fourth production round, including 14 esters, 12 alcohols, 6 acids, 5 nitrogenous compounds, 4 aldehydes and ketones, 3 phenolics, 3 furans, 2 lactones, and 1 compound from another chemical class. Among them, five compounds had FD values of 243, namely ethyl acetate (fruity, with baijiu aroma), ethyl furfuryl ether (sweet, spicy), furfuryl acetate (fruity), ethyl nicotinate (fruity, fumigate), and benzyl alcohol (fruity, rose). These odorants exhibited particularly persistent activity in the TF-4 AEDA extract and were further evaluated using quantitative analysis and OAVs. A total of four compounds appeared only in the fourth-round base baijiu, including methyl cyclopentenolone (nutty, licorice), phenol (barbecue), γ-decalactone (fruity, peach), and eugenol (clove). These odor-active compounds were detected specifically in TF-4 under the present analytical conditions.

A total of 53 odor-active compounds were detected in TF-7, including 13 esters, 11 alcohols, 7 acids, 9 nitrogenous compounds, 5 aldehydes and ketones, 4 furans, 1 phenolic, 1 lactone, 1 sulfur-containing compound, and 1 compound from another chemical class. There were seven compounds with an FD value of 243, namely 3-methyl-1-butanol (burnt, malty), furfuryl acetate (fruity), 2-ethyl-6-methylpyrazine (nutty, roasted), benzeneacetaldehyde (floral, apple), isovaleric acid (sour odor, cheese), ethyl phenylacetate (rose, honey), and phenylethyl alcohol (honey, clove). These compounds showed particularly persistent odor activity in the TF-7 AEDA extract and were subsequently assessed together with the quantitative and OAV results. A total of 11 constituents were uniquely detected in the seventh-round samples, namely ethyl butyrate (pineapple, fruity), 2-methylpyrazine (nutty, roasted), acetoin (butter, creamy), tetramethylpyrazine (baked), 2-acetylpyridine (popcorn, tobacco), 3-methylpentanoic acid (cheese, green), ethyl laurate (fruity, fat odor), geranylacetone (orange peel, lemon peel), 3-phenylfuran (caramel, nutty, burnt), S-methyl thiofuroate (floral, bean), and ethyl 3-phenylpropionate (fruity, rose). These odor-active compounds were detected specifically in TF-7 under the present analytical conditions.

Analysis using AEDA demonstrated that the three samples contained a wide range of aroma compounds. Specifically, 3-methyl-1-butanol, ethyl furfuryl ether, and phenylethyl alcohol showed FD values of 81 or higher in all samples, indicating consistently persistent odor activity during serial dilution of the corresponding AEDA extracts. Their sensory relevance was further evaluated using quantitative analysis, OAVs, and aroma recombination and omission experiments. Compounds exhibiting notable FD variation between rounds included ethyl acetate, 2-ethyl-6-methylpyrazine, 1-octen-3-ol, furfuryl acetate, (2,2-diethoxyethyl)-benzene, ethyl nicotinate, geraniol, benzyl alcohol, 4-ethyl-2-methoxyphenol, and γ-nonalactone. The observed differences in aroma-compound profiles and FD factors were associated with distinct odor profiles among the representative production rounds.

The odor profiles detected by AEDA differed among the three representative production rounds. TF-1 showed relatively prominent spicy, roasted, burnt, and bitter odor notes associated with several phenolic compounds and alcohols. TF-4 was characterized by a greater representation of fruity and floral odor notes associated with esters and alcohols, whereas TF-7 contained several pyrazines and organic acids associated with nutty, roasted, cheesy, and sweaty odor notes. These differences in odor profiles among production rounds are generally consistent with previous observations for Sauce-flavor baijiu [29].

### 3.4. Quantification and OAV Analysis of Aroma Compounds Across Production Rounds

#### 3.4.1. Quantification of Aroma Compounds by GC-MS/MS and GC-FID

The internal standard calibration method was used to quantify 70 aroma compounds selected on the basis of the AEDA-GC-O-MS results. Tables S1 and S2 summarize the calibration and method validation results for the high-content and low-content compounds, respectively. The calibration curves showed R^2^ values ranging from 0.9903 to 0.9999, while recoveries ranged from 85.01% to 114.78%. The intraday and interday RSDs were 1.51–7.42% and 2.56–8.93%, respectively. For the compounds quantified by GC-MS/MS, the LODs and LOQs ranged from 0.26 to 66.46 μg/L and from 0.85 to 221.54 μg/L, respectively. These validation results demonstrated satisfactory linearity, accuracy, and precision for both quantitative methods, while analytical sensitivity in terms of LOD and LOQ was additionally evaluated for the GC-MS/MS method. For analytes exceeding the upper calibration limit, samples were diluted before instrumental analysis, and the concentrations in the original samples were calculated using the corresponding dilution factors. All analyzed solutions were maintained within the validated calibration ranges, and no quantitative values were obtained by extrapolation beyond these ranges. Quantification results for high-content and low-content aroma compounds are presented in Tables S3 and S4.

**Figure 4:**
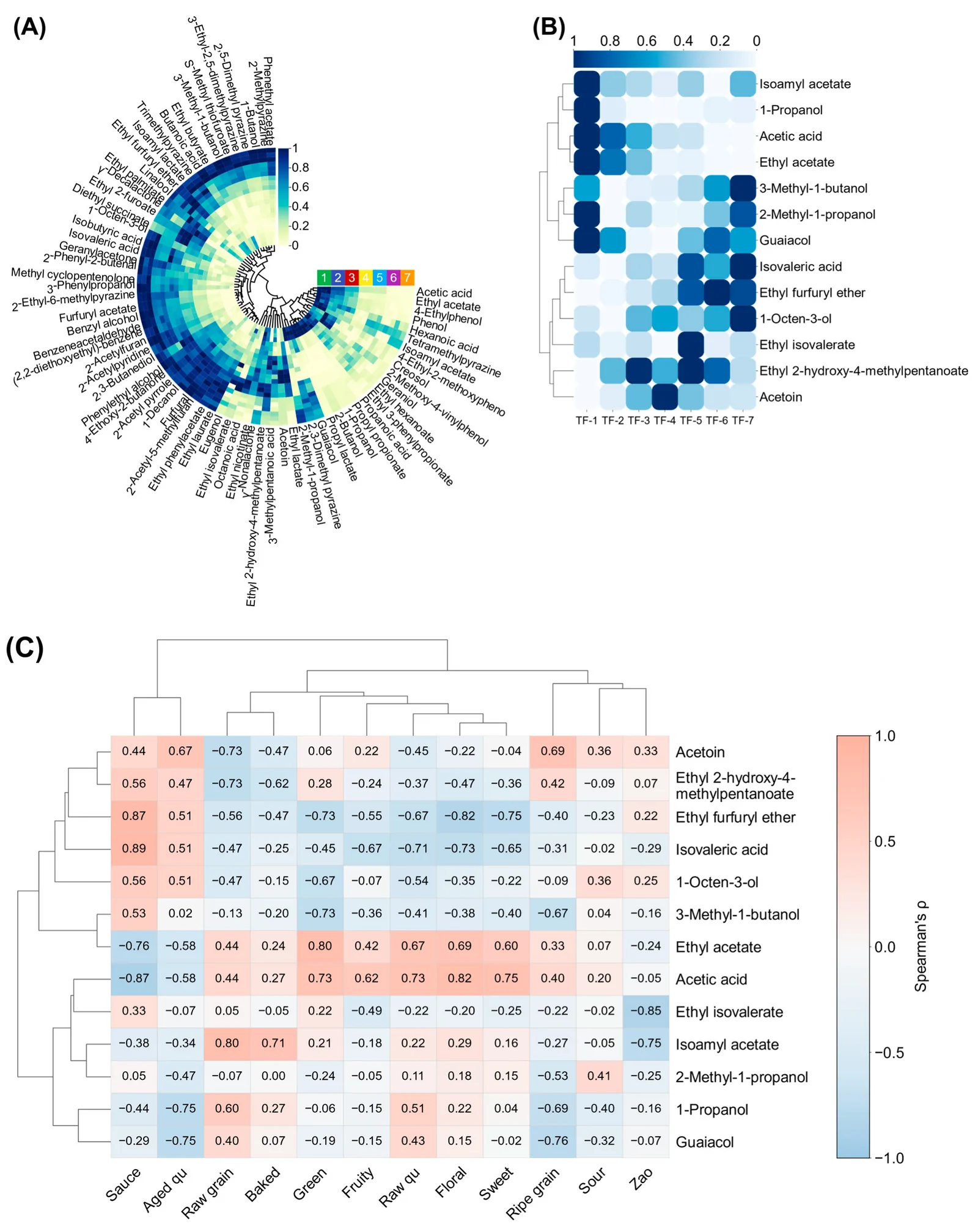
(**A**) Heatmap of normalized concentration patterns of 70 aroma compounds quantified by GC-FID and GC-MS/MS; (**B**) Heatmap of normalized concentration patterns of the 13 omission-supported aroma compounds across production rounds of Sauce-flavor base baijiu; (**C**) hierarchically clustered Spearman correlation heatmap showing associations between the 13 key aroma compounds and 12 sensory attributes across the seven production rounds (*n* = 7). Values within the cells represent Spearman’s rank correlation coefficients (ρ). Rows and columns were independently ordered by average-linkage hierarchical clustering based on Euclidean distances between correlation profiles.

Compounds with relatively high concentrations included 1-propanol (404.39–26,118.24 mg/L), ethyl acetate (1592.16–5018.87 mg/L), acetic acid (922.90–3080.95 mg/L), and ethyl lactate (1182.68–2838.41 mg/L). The highest alcohol content in base baijiu across production rounds was observed for 1-propanol (Figure 4A). It showed particularly high concentrations in the first and second production rounds, with values of 26,118.24 mg/L and 3289.82 mg/L, respectively, then gradually decreased, reaching a minimum of 404.39 mg/L in the fourth round, before increasing again in subsequent rounds. 1-propanol was the most abundant quantified higher alcohol, showing a pronounced round-dependent concentration pattern [30]. Acetic acid was the most abundant quantified acid and decreased progressively from 3080.95 mg/L in TF-1 to 922.90 mg/L in TF-7. The dominant esters in base baijiu across production rounds were ethyl acetate and ethyl lactate. While ethyl acetate steadily decreased from 5018.87 mg/L in the first round to 1592.16 mg/L in the seventh, ethyl lactate showed an initial increase, reaching its highest concentration of 2838.41 mg/L in the fourth round, and subsequently decreased to 1182.68 mg/L by the seventh round.

#### 3.4.2. Screening of Candidate Aroma-Active Compounds Based on OAVs

The OAVs of 70 aroma compounds were calculated by combining the quantitative results with odor thresholds reported in the literature (Table 3). Because these thresholds were not determined directly in the investigated baijiu matrices, the resulting OAVs should be interpreted as approximate screening indices rather than absolute measures of sensory contribution. Differences in ethanol concentration, nonvolatile matrix composition, and sensory testing conditions may influence odor release and perception and consequently alter the effective odor threshold. Among the three samples, 37 compounds showed literature-based OAV values ≥ 1 and were, therefore, selected as candidate aroma-active compounds for further sensory evaluation, consisting of 13 esters, 8 alcohols, 6 acids, 3 nitrogen-containing compounds, 3 aldehydes and ketones, 2 phenolics, 1 furan, and 1 lactone.

1-Propanol, which presented pungent and bitter notes, had the highest odor activity value in the first round of base baijiu, while the OAVs were reduced in rounds four and seven, attaining a minimum of 7 in the fourth round. In the fourth and seventh rounds, ethyl furfuryl ether, with sweet and spicy notes, displayed the highest OAV, consistent with the GC-O-MS results. It had the highest OAV value of 1081 in the seventh round, making it the only compound with an OAV value greater than 1000, suggesting potentially high odor relevance on the basis of the literature threshold. Its sensory contribution was further evaluated through the recombination and omission experiments.

Except for ethyl furfuryl ether and 1-propanol, ethyl isovalerate, acetoin, ethyl acetate, and isoamyl acetate showed calculated OAVs > 100 in at least one of the three samples, indicating that these compounds were priority candidates for sensory evaluation based on the literature thresholds. Ethyl isovalerate had OAV > 300 in all three samples; it has a high OAV value because of its low threshold and is considered a key aroma compound for Sauce-flavor base baijiu [12]. In addition, ethyl butyrate, ethyl hexanoate, isovaleric acid, and isobutyric acid had OAV values between 10 and 100 in all three samples. These compounds are associated with fruity, sour, and cheesy odor descriptors and were, therefore, retained as candidates for subsequent sensory evaluation. In total, eleven aroma-active compounds, such as ethyl lactate, benzeneacetaldehyde, acetic acid, 1-octen-3-ol, etc., had OAVs ranging from 10 to 100 in at least one of the three samples. These compounds were associated with fruity, sour, and baked odor descriptors and likewise remained candidates for subsequent sensory evaluation. The remaining sixteen compounds, including ethyl 2-hydroxy-4-methylpentanoate, guaiacol, and trimethylpyrazine, showed calculated OAVs below 10 but ≥1 and were, therefore, retained as candidate aroma-active compounds for subsequent sensory evaluation. Their sensory relevance was not inferred from OAV magnitude alone.

Furthermore, analyzing the differences in the OAV values of the base baijiu across production rounds revealed that compounds, such as ethyl furfuryl ether, 1-propanol, acetoin, ethyl acetate, isoamyl acetate, propyl propionate, 2-butanol, benzeneacetaldehyde, 1-butanol, furfural, phenylethyl alcohol, and ethyl laurate, had large differences in the OAV values. These compounds showed pronounced round-dependent differences in calculated OAVs. Overall, the OAV analysis was used as a screening approach rather than as definitive evidence of the sensory importance of individual compounds. Because the odor thresholds were obtained from the literature, the calculated OAV values may differ from those that would be obtained using thresholds measured directly in TF-1, TF-4, and TF-7, particularly because the samples differed in ethanol concentration and matrix composition. Therefore, the sensory relevance of candidate compounds was evaluated further using aroma recombination and omission experiments.

### 3.5. Validation of Key Aroma Compounds by Aroma Recombination and Aroma Omission Experiments

#### 3.5.1. Aroma Recombination of Key Aroma Compounds

Aroma recombination models containing 34, 35, and 37 compounds were constructed for TF-1, TF-4, and TF-7, respectively. The complete composition and final target concentrations of the aroma compounds included in the three recombination models are provided in Table S6. As shown in Figure 1B–D, most aroma attributes of the recombination models were similar to those of the corresponding original samples under the matched ethanol conditions. However, grain-related aroma was consistently weaker in the recombination models. This discrepancy may reflect contributions from compounds not included on the basis of the OAV criterion, interactions among aroma compounds, or differences between the simplified hydroalcoholic matrix and the original baijiu matrix. Overall, the recombination results indicate that the selected compounds collectively reproduced major sensory characteristics of TF-1, TF-4, and TF-7 under the model conditions. They do not demonstrate that every compound with OAV ≥ 1 contributes independently or that compounds with OAV < 1 are irrelevant.

#### 3.5.2. Omission Experiments of Key Aroma Compounds

Omission experiments were performed using the triangle test to evaluate whether removal of an individual compound produced a perceptible difference from the corresponding complete recombination model (Table S5). Of the compounds examined, 13 yielded significant sensory differences in at least one of the three models (Table 4), including ethyl furfuryl ether (OAV = 60–1081) and ethyl isovalerate (OAV = 386–477). Specifically, 1-propanol displayed a significant difference in the first-round omission model, with an OAV of 484 in the first round and OAVs < 50 in the other rounds. Omission of acetoin produced significant sensory differences in the TF-4 and TF-7 models, with OAVs of 329 and 70, respectively. Omission of ethyl acetate, isoamyl acetate, and acetic acid produced significant differences only in the TF-1 model, whereas isovaleric acid and 2-methyl-1-propanol were significant only in the fourth-round model. Although ethyl 2-hydroxy-4-methylpentanoate, guaiacol, and 3-methyl-1-butanol had OAVs below 10, they still exhibited significant differences across the omission models. These results demonstrate that OAV provides a useful initial criterion for selecting candidate aroma-active compounds, whereas sensory validation remains necessary to determine the perceptible effect of individual compounds within the recombination model. Notably, several compounds with relatively low OAVs produced significant omission effects, indicating that OAV magnitude alone does not fully predict sensory relevance.

### 3.6. Variation and Possible Origins of Key Aroma Compounds Across Production Rounds

The concentration trajectories of the 13 compounds that produced significant omission effects were examined across the seven production rounds (Figure 4B). The concentrations of 1-propanol, 3-methyl-1-butanol, and 2-methyl-1-propanol generally decreased during the early production rounds and increased again in the later rounds, with 1-propanol showing a particularly high concentration in TF-1. Previous studies have associated higher alcohol formation in baijiu with microbial carbohydrate and amino acid metabolism [27]. Notably, metagenomic and metabolomic analyses of the first two production rounds of Sauce-flavor baijiu demonstrated substantially greater 1-propanol accumulation during the first round and revealed microbial and metabolic features associated with its formation [31]. Moreover, substantial changes in microbial communities, starch, reducing sugars, and flavor metabolites have been observed across the seven fermentation rounds of Sauce-flavor baijiu [27]. Therefore, the variation in higher alcohol concentrations observed across production rounds may be related to changes in microbial metabolism and substrate availability reported previously. However, microbial communities, carbohydrate and amino acid concentrations, and metabolic intermediates were not measured in the present study; therefore, these mechanisms remain possible explanations rather than processes demonstrated by the present dataset.

Ethyl acetate decreased progressively across production rounds, whereas isoamyl acetate decreased initially and increased again in TF-7. Ethyl isovalerate and ethyl 2-hydroxy-4-methylpentanoate exhibited different round-dependent patterns. Ester formation in baijiu involves multiple biochemical processes associated with microbial fatty acid and amino acid metabolism, together with chemical esterification and hydrolysis reactions [32]. Previous studies of Sauce-flavor baijiu have further shown that changes in microbial communities across production rounds are associated with variations in ester and other volatile compound profiles [17,18]. Therefore, in the context of these previous findings, the different trends observed for these esters may reflect round-dependent changes in precursor availability and microbial metabolism; however, the relative contributions of biosynthesis, esterification, and hydrolysis cannot be resolved from the present measurements. Although these esters are generally associated with fruity aroma notes, ethyl acetate can also impart pungent and solvent-like notes at elevated concentrations, whereas ethyl isovalerate and ethyl 2-hydroxy-4-methylpentanoate are characterized by fruity and sweet odor attributes. Together with the omission-test results, these concentration changes suggest that the relative abundance of individual esters contributes differently to the aroma profiles of the investigated production rounds.

Acetic acid decreased progressively across production rounds, whereas isovaleric acid generally increased during the later rounds. Previous studies have reported associations between organic acid profiles, microbial community changes, and substrate availability during Sauce-flavor baijiu fermentation [27,33]. A study covering seven fermentation rounds demonstrated substantial changes in microbial composition, starch, reducing sugars, acidity, and organic acids [27], while analyses of microorganisms and metabolites reported associations between acetic acid, isobutyric acid, isovaleric acid, and specific microbial taxa and metabolic pathways [33]. Thus, the different trends of acetic acid and isovaleric acid observed in the present study may be interpreted in the context of these previously reported changes in acid metabolism. Because microbial communities, substrates, amino acids, and metabolic intermediates were not measured here, processes such as substrate depletion, proteolysis, amino acid conversion, and microbial succession cannot be assigned as direct causes of the observed acid variation.

From the perspective of precursor origin, previous studies of Sauce-flavor baijiu indicate that sorghum provides major carbohydrate, nitrogenous, lipid, and phenolic substrates, while high-temperature wheat Daqu supplies microorganisms, enzymes, and additional aroma precursors [5,34]. During fermentation, starch-derived carbohydrates can be converted into ethanol and organic acids, while amino acids provide precursors for higher alcohols, aldehydes, and acids. Fatty acids and alcohols can further contribute to ester formation through microbial metabolism and esterification reactions [32]. In addition, transformations occurring during high-temperature Daqu production and fermentation can generate aromatic and heterocyclic compounds through microbial metabolism and heat-related reactions [35]. These pathways provide a possible framework for understanding the origins and transformations of the aroma compounds detected in the present study. However, raw-material composition, precursor concentrations, enzyme activities, and metabolic intermediates were not measured; therefore, these pathways should be regarded as explanations supported by the literature rather than mechanisms demonstrated here.

Among the remaining key compounds, 1-octen-3-ol and ethyl furfuryl ether generally increased across rounds, acetoin increased initially and subsequently decreased, whereas guaiacol showed a non-monotonic pattern. These compound-specific trajectories demonstrate distinct round-dependent patterns of aroma-compound accumulation. Because the precursors and formation pathways of these compounds were not investigated directly, no specific formation mechanism is assigned to these variations in the present study.

### 3.7. Associations Between Key Aroma Compounds and Sensory Attributes

Spearman rank correlation analysis was further conducted to explore the co-variation between the concentrations of the 13 key aroma compounds and the 12 sensory attributes across the seven production rounds (Figure 4C). Analytical replicates were averaged within each production round for each aroma compound, while the sensory scores of the ten assessors were averaged within each production round for each attribute. Thus, the seven production rounds were treated as the effective observations (*n* = 7). Given the limited effective sample size, the correlation coefficients were interpreted descriptively as measures of the direction and magnitude of monotonic co-variation rather than as inferential evidence of direct sensory effects. After Benjamini–Hochberg correction for the 156 compound–attribute comparisons, none of the associations remained statistically significant at an adjusted *p* < 0.05 (minimum adjusted *p* = 0.453). Accordingly, the correlation coefficients below are discussed descriptively as exploratory co-variation patterns rather than as statistically confirmed compound–sensory relationships.

Several relatively strong associations were observed. Sauce aroma showed positive associations with isovaleric acid (ρ = 0.89) and ethyl furfuryl ether (ρ = 0.87), whereas negative associations were observed with acetic acid (ρ = −0.87) and ethyl acetate (ρ = −0.76). Floral aroma showed contrasting association patterns with ethyl furfuryl ether (ρ = −0.82) and acetic acid (ρ = 0.82). Raw grain aroma was positively associated with isoamyl acetate (ρ = 0.80), whereas green aroma showed a positive association with ethyl acetate (ρ = 0.80). In addition, ethyl isovalerate was negatively associated with Zao aroma (ρ = −0.85), and guaiacol showed a negative association with ripe-grain aroma (ρ = −0.76).

These compound–sensory co-variation patterns are broadly consistent with previous studies showing that the sensory characteristics of Jiang-flavor baijiu are closely associated with variations in the composition and abundance of key aroma compounds [4,11]. Previous sensory–flavoromics investigations have likewise demonstrated substantial covariation between volatile composition and sensory profiles among Sauce-flavor baijiu samples from different origins [20]. Moreover, marked differences in flavor characteristics among successive production rounds have previously been reported using complementary instrumental and sensory approaches [29]. The present correlation analysis extends these observations by describing how selected key aroma compounds and sensory attributes co-varied across the seven production rounds investigated here.

Hierarchical clustering of the correlation matrix further grouped aroma compounds and sensory attributes according to similarities in their overall correlation profiles. The clustering was used to facilitate visualization of related co-variation patterns and should not be interpreted as evidence of common formation pathways or direct sensory interactions.

Overall, the Spearman analysis revealed distinct positive and negative associations between key aroma-compound concentrations and sensory attributes across production rounds. However, because the analysis was based on only seven effective observations from a single production sequence and involved a large number of compound–attribute comparisons, these associations should be regarded as exploratory. They do not establish causal, synergistic, antagonistic, enhancing, masking, or suppressing effects. The sensory relevance of individual compounds was, therefore, evaluated primarily on the basis of the aroma recombination and omission experiments, while the correlation analysis provided complementary information on round-dependent co-variation.

## 4. Conclusions

In this work, aroma compounds of Sauce-flavor base baijiu from seven production rounds in the Tianfu region were systematically characterized using QDA, GC-MS profiling, AEDA-GC-O-MS, quantitative GC-FID and GC-MS/MS analyses, OAV screening, and aroma recombination and omission experiments. A total of 70 compounds were quantified, and 37 showed literature-based OAV values ≥ 1 and were selected as candidate aroma-active compounds for further sensory evaluation. Quantitative analysis revealed distinct concentration patterns across production rounds, while aroma recombination and omission experiments provided further sensory evidence for selected compounds. Within the investigated production system, these findings provide a quantitative and sensory characterization of aroma variation across successive production rounds and may offer a reference for flavor regulation, base baijiu grading, and blending optimization. However, the samples originated from a single production source in the Tianfu region and represented successive stages within one production sequence, with only analytical replication available within each stage. Therefore, the observed aroma patterns and candidate aroma compounds should be considered specific to the investigated system and should not be assumed to be generalizable to other Sauce-flavor baijiu producers, geographical regions, or production systems. Further studies using independent production batches from multiple production sources and geographical contexts are required to evaluate the broader reproducibility and practical applicability of these findings.

## Figures and Tables

**Table 1:** SRM acquisition parameters for GC–MS/MS quantification.

Aroma Compounds	CAS	Retention Time (min)	SRM Transition (*m*/*z*); Collision Energy (eV)			
Ethyl butyrate	105-54-4	6.83	71.00/43.10; 5 *	71.00/41.10; 10	88.00/60.00; 10	88.00/61.00; 5
Propyl propionate	106-36-5	6.92	75.00/57.00; 5 *	57.00/29.10; 5	75.00/29.10; 15	87.00/29.10; 10
Ethyl isovalerate	108-64-5	7.2	85.00/57.10; 5 *	70.00/55.00; 5	85.00/41.10; 10	88.00/45.00; 10
Isoamyl acetate	123-92-2	7.94	70.00/55.00; 5 *	61.00/43.00; 10	70.00/29.10; 15	70.00/42.10; 5
Ethyl hexanoate	123-66-0	9.45	101.00/73.00; 5 *	88.00/60.00; 10	88.00/61.00; 5	99.00/43.10; 10
4-Ethoxy-2-butanone	60044-74-8	10.28	72.00/43.00; 5 *	59.00/31.00; 5	72.00/57.00; 5	87.00/43.00; 5
Ethyl furfuryl ether	6270-56-0	10.5	81.00/53.00; 10 *	82.00/39.10; 15	82.00/54.10; 10	82.00/81.00; 5
2-Methylpyrazine	109-08-0	10.58	53.00/26.10; 5 *	67.00/26.10; 10	67.00/41.10; 5	67.00/42.10; 10
Acetoin	513-86-0	11	73.00/45.00; 5 *	45.00/27.10; 15	45.00/29.00; 10	45.00/41.00; 35
2,5-Dimethyl pyrazine	123-32-0	11.5	108.00/81.10; 10 *	81.00/39.00; 20	81.00/42.00; 5	107.00/80.00; 10
2,3-Dimethyl pyrazine	5910-89-4	12	108.00/67.10; 10 *	67.00/26.10; 10	67.00/40.10; 5	67.00/41.10; 5
2-Ethyl-6-methylpyrazine	13925-03-6	12.7	122.00/121.10; 10 *	94.00/41.10; 10	94.00/66.00; 10	121.00/53.00; 15
Trimethylpyrazine	14667-55-1	13.15	122.00/81.10; 10 *	81.00/42.10; 5	122.00/42.10; 15	122.00/80.10; 10
Propyl lactate	616-09-1	13.45	75.00/45.00; 5 *	41.00/39.10; 10	43.00/41.10; 5	45.00/27.10; 15
2-propylpyrazine (IS1)	18138-03-9	13.48	122.00/94.00; 10 *	94.00/39.00; 25	94.00/67.10; 10	94.00/93.00; 10
1-Octen-3-ol	3391-86-4	13.74	72.00/43.00; 5 *	57.00/29.10; 5	57.00/31.00; 15	72.00/57.00; 5
3-Ethyl-2,5-dimethylpyrazine	13360-65-1	14	136.00/135.10; 10 *	108.00/107.10; 5	135.00/80.00; 15	135.00/107.10; 10
Tetramethylpyrazine	1124-11-4	14.8	136.00/54.10; 15 *	135.00/94.10; 10	136.00/39.10; 25	136.00/94.10; 10
2-Acetylfuran	1192-62-7	15.8	110.00/95.00; 5 *	67.00/39.00; 5	95.00/38.10; 35	95.00/39.10; 15
Furfuryl acetate	623-17-6	16.36	140.00/98.00; 5 *	81.00/53.00; 10	98.00/42.10; 10	98.00/69.00; 15
Linalool	78-70-6	16.41	93.00/91.10; 5 *	93.00/77.00; 10	107.00/91.10; 10	121.00/77.00; 20
Ethyl 2-hydroxy-4-methylpentanoate	10348-47-7	16.5	87.00/69.10; 5 *	69.00/41.10; 10	87.00/41.10; 10	87.00/43.10; 10
Isoamyl lactate	19329-89-6	17.32	70.00/55.10; 10 *	55.00/29.10; 10	70.00/42.10; 5	71.00/41.10; 10
Isobutyric acid	79-31-2	17.34	88.00/73.00; 5 *	60.00/42.00; 10	73.00/27.10; 20	73.00/55.00; 10
2-Acetylpyridine	1122-62-9	18.78	121.00/93.10; 5 *	78.00/51.00; 10	79.00/51.10; 20	79.00/52.00; 10
2-Acetyl-5-methylfuran	1193-79-9	19.07	124.00/109.00; 10 *	81.00/53.00; 5	109.00/53.00; 10	110.00/53.10; 10
Ethyl 2-furoate	614-99-3	19.13	140.00/112.00; 5 *	95.00/67.00; 10	96.00/95.00; 10	112.00/84.00; 10
Butanoic acid	107-92-6	19.27	60.00/42.00; 10 *	45.00/41.00; 15	55.00/27.10; 10	60.00/45.00; 10
Benzeneacetaldehyde	122-78-1	20.08	120.00/92.10; 5 *	65.00/39.00; 10	91.00/39.00; 25	91.00/65.00; 10
Isovaleric acid	503-74-2	20.556	87.00/69.00; 5 *	60.00/42.00; 10	60.00/45.00; 15	69.00/41.10; 5
Diethyl succinate	123-25-1	20.62	129.00/101.00; 5 *	101.00/45.00; 15	101.00/55.00; 5	101.00/73.00; 5
(2,2-diethoxyethyl)-benzene	6314-97-2	22.1	121.00/103.00; 10 *	91.00/65.00; 15	103.00/47.00; 10	103.00/75.10; 5
1-Decanol	112-30-1	23.31	111.00/69.10; 5 *	70.00/42.10; 5	70.00/55.10; 5	83.00/41.10; 15
Ethyl phenylacetate	101-97-3	24.52	164.00/91.10; 10 *	65.00/39.00; 10	91.00/39.00; 25	91.00/65.00; 10
3-Methylpentanoic acid	105-43-1	24.64	87.00/69.00; 5 *	57.00/41.10; 5	60.00/42.00; 10	60.00/45.00; 15
Phenethyl acetate	103-45-7	25.55	104.00/78.00; 10 *	91.00/65.00; 10	103.00/77.00; 15	104.00/77.00; 25
Ethyl nicotinate	614-18-6	25.62	106.00/78.00; 10 *	106.00/51.00; 20	123.00/77.00; 15	123.00/105.00; 10
Methyl cyclopentenolone	80-71-7	25.95	112.00/84.10; 5 *	69.00/39.00; 15	69.00/41.10; 5	84.00/55.10; 10
Ethyl laurate	106-33-2	26.1	101.00/73.00; 5 *	88.00/61.00; 5	101.00/55.00; 15	157.00/87.10; 10
Geraniol	106-24-1	26.22	111.00/93.10; 5 *	69.00/39.00; 15	69.00/41.10; 5	93.00/77.10; 10
Hexanoic acid	142-62-1	26.4	73.00/55.00; 10 *	60.00/42.00; 10	60.00/45.00; 10	87.00/43.00; 5
Geranylacetone	3796-70-1	26.67	136.00/121.10; 5 *	107.00/91.10; 10	136.00/93.10; 10	151.00/77.00; 25
S-Methyl thiofuroate	13679-61-3	26.77	142.00/95.00; 10 *	67.00/39.00; 5	95.00/38.10; 35	95.00/39.00; 15
Guaiacol	90-05-1	27.06	124.00/109.00; 10 *	81.00/53.00; 10	109.00/53.10; 15	109.00/81.00; 10
Benzyl alcohol	100-51-6	27.54	108.00/107.00; 10 *	77.00/51.00; 15	79.00/77.00; 10	107.00/77.00; 20
Ethyl 3-phenylpropionate	2021-28-5	27.8	178.00/104.10; 5 *	107.00/79.10; 10	108.00/80.10; 10	133.00/105.10; 5
Phenylethyl alcohol	60-12-8	28.71	122.00/92.10; 5 *	91.00/39.00; 25	91.00/65.00; 15	92.00/65.00; 20
2-Phenyl-2-butenal	4411-89-6	29.54	146.00/117.10; 5 *	91.00/65.00; 10	115.00/89.00; 20	117.00/91.10; 15
Creosol	93-51-6	30.23	138.00/123.10; 10 *	95.00/67.10; 6	123.00/67.00; 10	123.00/95.00; 10
2-Acetyl pyrrole	1072-83-9	30.738	109.00/94.00; 10 *	66.00/38.10; 30	66.00/39.00; 10	94.00/39.10; 20
Phenol	108-95-2	31.882	94.00/66.10; 10 *	66.00/39.10; 15	66.00/40.10; 10	66.00/65.00; 5
4-Ethyl-2-methoxyphenol	2785-89-9	32.597	152.00/137.10; 10 *	122.00/94.00; 5	137.00/65.10; 30	137.00/66.10; 25
γ-Nonalactone	104-61-0	32.744	85.00/57.00; 5 *	85.00/27.10; 20	85.00/29.10; 10	100.00/43.00; 10
3-Phenylpropanol	122-97-4	33.03	118.00/117.10; 10 *	117.00/65.00; 30	117.00/91.00; 20	117.00/115.10; 15
Octanoic acid	124-07-2	33.4	73.00/55.00; 10 *	60.00/42.00; 10	101.00/57.00; 5	101.00/73.10; 5
γ-Decalactone	706-14-9	36.36	85.00/57.00; 5 *	85.00/27.10; 20	85.00/29.10; 10	110.00/95.10; 5
Eugenol	97-53-0	36.9	164.00/149.10; 10 *	131.00/103.10; 10	137.00/122.00; 10	149.00/121.10; 5
4-Ethylphenol	123-07-9	37.17	122.00/107.00; 10 *	77.00/51.00; 15	107.00/51.00; 25	107.00/77.00; 15
2-Methoxy-4-vinylphenol	7786-61-0	37.83	150.00/135.10; 10 *	107.00/77.00; 10	135.00/77.00; 15	135.00/107.00; 5
3,4-dimethylphenol (IS2)	95-65-8	38.48	122.00/107.10; 10 *	107.00/51.00; 25	107.00/77.00; 15	107.00/79.10; 10

*: Quantitative transitions and corresponding collision energies.

**Table 2:** Odor-active compounds characterized by AEDA-GC-O-MS in representative Sauce-flavor base baijiu production rounds.

No.	Aroma Compounds	CAS	Odor Descriptor ^a^	FD ^b^			RI ^c^	Identification Basis ^d^
				TF-1	TF-4	TF-7		
1	Ethyl acetate	141-78-6	fruity, with baijiu aroma	243	243	27	891	MS, RI, Odor, Std
2	2-Butanol	78-92-2	wine	81	3	27	1022	MS, RI, Odor, Std
3	1-Propanol	71-23-8	pungent, bitter	81	9	81	1038	MS, RI, Odor, Std
4	Ethyl butyrate	105-54-4	pineapple, fruity	ND	ND	81	1041	MS, RI, Odor, Std
5	Propyl propionate	106-36-5	apple, banana	27	ND	ND	1045	MS, RI, Odor, Std
6	Ethyl isovalerate	108-64-5	apple, pineapple	27	27	ND	1072	MS, RI, Odor, Std
7	2-Methyl-1-propanol	78-83-1	apple, bitter	9	81	81	1101	MS, RI, Odor, Std
8	Isoamyl acetate	123-92-2	apple, pear	3	9	9	1136	MS, RI, Odor, Std
9	1-Butanol	71-36-3	fruity	9	1	1	1150	MS, RI, Odor, Std
10	3-Methyl-1-butanol	123-51-3	burnt, malty	243	81	243	1208	MS, RI, Odor, Std
11	Ethyl hexanoate	123-66-0	apple peel, brandy	1	9	1	1245	MS, RI, Odor, Std
12	4-Ethoxy-2-butanone	60044-74-8	fruity	3	ND	ND	1276	MS, RI, Odor, Std
13	2-Methylpyrazine	109-08-0	nutty, roasted	ND	ND	3	1288	MS, RI, Odor, Std
14	Ethyl furfuryl ether	6270-56-0	sweet, spicy	81	243	243	1295	MS, RI, Odor, Std
15	Acetoin	513-86-0	butter, creamy	ND	ND	9	1304	MS, RI, Odor, Std
16	Ethyl lactate	97-64-3	fruity	1	3	1	1341	MS, RI, Odor, Std
17	2,5-Dimethyl pyrazine	123-32-0	fried peanuts, creamy	3	3	81	1344	MS, RI, Odor, Std
18	2,3-Dimethyl pyrazine	5910-89-4	roasted, hazel	1	3	1	1375	MS, RI, Odor, Std
19	2-Ethyl-6-methylpyrazine	13925-03-6	nutty, roasted	27	27	243	1404	MS, RI, Odor, Std
20	Trimethylpyrazine	14667-55-1	potato, roasted	27	ND	3	1419	MS, RI, Odor, Std
21	Propyl lactate	616-09-1	fruity, grape	1	3	ND	1439	MS, RI, Odor, Std
22	1-Octen-3-ol	3391-86-4	mushroom	1	1	81	1447	MS, RI, Odor, Std
23	Acetic acid	64-19-7	acid, vinegar	9	3	3	1451	MS, RI, Odor, Std
24	Isobutyl lactate	585-24-0	butter, caramel	81	ND	ND	1456	MS, Odor
25	3-Ethyl-2,5-dimethylpyrazine	13360-65-1	roasted	ND	3	3	1459	MS, RI, Odor, Std
26	2,6-Diethylpyrazine	13067-27-1	baked, soil	3	ND	ND	1467	MS, Odor
27	Furfural	98-01-1	almond, baked potatoes	27	27	27	1475	MS, RI, Odor, Std
28	Tetramethylpyrazine	1124-11-4	baked	ND	ND	1	1482	MS, RI, Odor, Std
29	2-Methyl-4-octanol	40575-41-5	fruity, floral	3	ND	ND	1493	MS, Odor
30	2-Acetylfuran	1192-62-7	nutty, roasted	1	3	9	1525	MS, RI, Odor, Std
31	Propanoic acid	79-09-4	sour odor, sweat	9	ND	ND	1534	MS, RI, Odor, Std
32	Furfuryl acetate	623-17-6	fruity	ND	243	81	1541	MS, RI, Odor, Std
33	Ethyl 2-hydroxy-4-methylpentanoate	10348-47-7	blackberry, fruity	27	9	ND	1545	MS, RI, Odor, Std
34	Linalool	78-70-6	ether, lemon	1	ND	9	1548	MS, RI, Odor, Std
35	Isobutyric acid	79-31-2	cheese, sweat	9	9	3	1567	MS, RI, Odor, Std
36	2,3-Butanediol	513-85-9	fruity, creamy	9	9	9	1577	MS, RI, Odor, Std
37	Isoamyl lactate	19329-89-6	fruity, nutty	1	1	1	1579	MS, RI, Odor, Std
38	Butanoic acid	107-92-6	sour odor, cheese	27	9	27	1627	MS, RI, Odor, Std
39	2-Acetylpyridine	1122-62-9	popcorn, tobacco	ND	ND	1	1627	MS, RI, Odor, Std
40	2-Acetyl-5-methylfuran	1193-79-9	roasted, cookie, almond	ND	1	3	1634	MS, RI, Odor, Std
41	Ethyl 2-furoate	614-99-3	kiwifruit, mushroom	ND	3	27	1637	MS, RI, Odor, Std
42	Benzeneacetaldehyde	122-78-1	floral, apple	27	81	243	1664	MS, RI, Odor, Std
43	Isovaleric acid	503-74-2	sour odor, cheese	27	81	243	1670	MS, RI, Odor, Std
44	Diethyl succinate	123-25-1	fruity, slightly sweet	9	ND	ND	1682	MS, RI, Odor, Std
45	2,5-Dimethyl-3-butylpyrazine	40790-29-2	roasted, nutty	ND	1	9	1715	MS, Odor
46	(2,2-diethoxyethyl)-benzene	6314-97-2	almond, orange blossom	1	3	81	1728	MS, RI, Odor, Std
47	1-Decanol	112-30-1	green	ND	9	9	1764	MS, RI, Odor, Std
48	Ethyl phenylacetate	101-97-3	rose, honey	27	27	243	1799	MS, RI, Odor, Std
49	3-Methylpentanoic acid	105-43-1	cheese, green	ND	ND	1	1803	MS, RI, Odor, Std
50	Phenethyl acetate	103-45-7	floral, honey	9	3	9	1829	MS, RI, Odor, Std
51	Ethyl nicotinate	614-18-6	fruity, fumigate	9	243	ND	1832	MS, RI, Odor, Std
52	Methyl cyclopentenolone	80-71-7	nutty, licorice	ND	3	ND	1842	MS, RI, Odor, Std
53	Ethyl laurate	106-33-2	fruity, fat odor	ND	ND	9	1846	MS, RI, Odor, Std
54	Geraniol	106-24-1	rose, lemon peel	81	27	ND	1851	MS, RI, Odor, Std
55	Hexanoic acid	142-62-1	cheese, Daqu	ND	9	1	1855	MS, RI, Odor, Std
56	Geranylacetone	3796-70-1	orange peel, lemon peel	ND	ND	9	1863	MS, RI, Odor, Std
57	3-Phenylfuran	13679-41-9	caramel, nutty, burnt	ND	ND	3	1871	MS, Odor
58	S-Methyl thiofuroate	13679-61-3	floral, bean	ND	ND	9	1865	MS, RI, Odor, Std
59	Guaiacol	90-05-1	burnt, wood	27	ND	ND	1873	MS, RI, Odor, Std
60	Benzyl alcohol	100-51-6	fruity, rose	81	243	1	1888	MS, RI, Odor, Std
61	Ethyl 3-phenylpropionate	2021-28-5	fruity, rose	ND	ND	9	1897	MS, RI, Odor, Std
62	Phenylethyl alcohol	60-12-8	honey, clove	81	81	243	1924	MS, RI, Odor, Std
63	2-Phenyl-2-butenal	4411-89-6	mildew, floral	ND	3	9	1949	MS, RI, Odor, Std
64	Creosol	93-51-6	fumigate, clove	9	ND	ND	1969	MS, RI, Odor, Std
65	2-Acetyl pyrrole	1072-83-9	nutty, brew	3	ND	ND	1985	MS, RI, Odor, Std
66	Phenol	108-95-2	roasted	ND	1	ND	2022	MS, RI, Odor, Std
67	4-Ethyl-2-methoxyphenol	2785-89-9	fumigate, roasted	81	ND	ND	2043	MS, RI, Odor, Std
68	γ-Nonalactone	104-61-0	coconut	243	3	9	2048	MS, RI, Odor, Std
69	3-Phenylpropanol	122-97-4	floral, sweet	81	9	ND	2058	MS, RI, Odor, Std
70	Octanoic acid	124-07-2	fat odor	3	27	27	2064	MS, RI, Odor, Std
71	4-Phenyl-3-buten-2-ol	17488-65-2	fruity, floral	9	ND	ND	2088	MS, Odor
72	γ-Decalactone	706-14-9	fruity, peach	ND	9	ND	2163	MS, RI, Odor, Std
73	Eugenol	97-53-0	clove	ND	81	ND	2180	MS, RI, Odor, Std
74	4-Ethylphenol	123-07-9	leather	27	27	81	2189	MS, RI, Odor, Std
75	2-Methoxy-4-vinylphenol	7786-61-0	spice, fried peanuts	9	ND	ND	2212	MS, RI, Odor, Std
76	Ethyl palmitate	628-97-7	oil	9	27	27	2258	MS, RI, Odor, Std

^a^ Odor attributes perceived at the GC-O sniffing port. ^b^ FD factors were determined by AEDA on a capillary TG-WAXMS column; ND: not detected at the sniffing port. ^c^ Retention indices (RIs) were calculated on the TG-WAXMS column using the C_7_–C_40_ n-alkane series. ^d^ Identification basis: MS, mass spectral information; RI, retention index on the TG-WAXMS column; Odor, consensus odor descriptor obtained at the sniffing port; Std, comparison with an authentic standard. Compounds identified using MS, RI, Odor, and Std were considered confirmed identifications, whereas compounds identified using MS and Odor without authentic standard confirmation were considered tentative identifications.

**Table 3:** OAV values of aroma compounds in Sauce-flavor base baijiu across representative production rounds.

No. ^a^	Aroma Compounds	Threshold ^b^ (μg/L)	OAV ^c^		
			TF-1	TF-4	TF-7
14	Ethyl furfuryl ether	30.89	60	394	1081
6	Ethyl isovalerate	6.89	477	386	471
3	1-Propanol	53,952.63	484	7	42
15	Acetoin	259	13	329	70
1	Ethyl acetate	32,551.6	154	54	49
8	Isoamyl acetate	93.93	149	29	77
4	Ethyl butyrate	81.5	42	25	90
11	Ethyl hexanoate	55.33	30	16	15
43	Isovaleric acid	1050	30	31	44
35	Isobutyric acid	1580	16	19	26
16	Ethyl lactate	128,000	12	22	9
23	Acetic acid	160,000	19	9	6
7	2-Methyl-1-propanol	28,300	11	5	10
38	Butanoic acid	964	7	7	21
22	1-Octen-3-ol	6.12	5	11	18
25	3-Ethyl-2,5-dimethylpyrazine	8.6	5	5	11
48	Ethyl phenylacetate	406.83	5	13	18
9	1-Butanol	2733.35	4	5	32
42	Benzeneacetaldehyde	262	4	16	35
31	Propanoic acid	18,100	11	2	2
19	2-Ethyl-6-methylpyrazine	40	3	8	19
33	Ethyl 2-hydroxy-4-methylpentanoate	1268	6	9	7
59	Guaiacol	13.4	6	2	4
5	Propyl propionate	880	9	<1	1
2	2-Butanol	50,000	8	<1	1
10	3-Methyl-1-butanol	179,000	2	1	3
20	Trimethylpyrazine	730	2	1	2
61	Ethyl 3-phenylpropionate	125.21	2	1	1
76	Ethyl palmitate	39,299.35	2	2	2
34	Linalool	25	1	2	2
50	Phenethyl acetate	909	1	1	4
55	Hexanoic acid	2517.16	1	1	1
68	γ-Nonalactone	91	1	1	1
73	Eugenol	21	1	1	1
27	Furfural	44,000	<1	6	8
53	Ethyl laurate	500	<1	1	1
62	Phenylethyl alcohol	28,922.73	<1	1	1

^a^ Same serial number as aroma compounds in Table 2. ^b^ Odor threshold previously reported in ref [5]. ^c^ The OAV value is calculated by dividing the concentration by the odor threshold of the aroma compound.

**Table 4:** Significant aroma omission test results for representative Sauce-flavor base baijiu production rounds.

No. ^a^	Aroma Compounds Omitted from the Recombination Model	Odor Descriptor	Significance ^b^		
			TF-1	TF-4	TF-7
1	Ethyl furfuryl ether	sweet, spicy	**	*	***
2	Ethyl isovalerate	apple, pineapple	***	**	**
3	1-Propanol	pungent, bitter	*	-	-
4	Acetoin	butter, creamy	-	**	*
5	Ethyl acetate	fruity, with baijiu aroma	***	-	-
6	Isoamyl acetate	apple, pear	*	-	-
9	Isovaleric acid	sour odor, cheese	-	***	-
12	Acetic acid	acid, vinegar	**	-	-
13	2-Methyl-1-propanol	apple, bitter	-	**	-
15	1-Octen-3-ol	mushroom	-	-	*
22	Ethyl 2-hydroxy-4-methylpentanoate	blackberry, fruity	**	-	-
23	Guaiacol	burnt, wood	-	-	**
26	3-Methyl-1-butanol	burnt, malty	-	-	**

^a^ Same serial number as aroma compounds in Table S5. ^b^ Significance: *, significant (*p*  <  0.05); **, highly significant (*p*  <  0.01); ***, very highly significant (*p*  <  0.001); -, not significant.

## Data Availability

The original contributions presented in the study are included in the article/Supplementary Materials. Further inquiries can be directed to the corresponding authors.

## Supplementary Materials

The following supporting information can be downloaded at https://www.mdpi.com/article/10.3390/foods15203698/s1, Figure S1, Distribution of volatile compounds tentatively identified by GC-MS based on NIST 2020 library matching in Sauce-flavor base baijiu across seven production rounds; Table S1, Method validation results for high-content aroma compounds; Table S2, Method validation results for low-content aroma compounds; Table S3, Quantitative results of high-content aroma compounds (mg/L); Table S4, Quantitative results of low-content aroma compounds (μg/L); Table S5, Aroma omission test results for Sauce-flavor base baijiu from representative production rounds; Table S6, Composition and final concentrations of aroma compounds included in the recombination models of TF-1, TF-4, and TF-7; Table S7, Raw sensory intensity scores assigned by ten trained assessors to 12 aroma attributes of Sauce-flavor base baijiu across seven production rounds.

## Abbreviations

The following abbreviations are used in this manuscript:

LLE	Liquid–liquid extraction
HS-SPME	Headspace solid phase microextraction
GC-MS	Gas chromatography–mass spectrometry
GC-O-MS	Gas chromatography coupled with olfactometry and mass spectrometry
GC-FID	Gas chromatography–flame ionization detection
GC-MS/MS	Gas chromatography–tandem mass spectrometry
SIM	Selected ion monitoring
SRM	Selected reaction monitoring
OAV	Odor activity value
AEDA	Aroma extract dilution analysis
FD	Flavor dilution factor
NBF	Neutral/basic fraction
AF	Acidic fraction
QDA	Quantitative descriptive analysis
OPLS-DA	Orthogonal partial least-squares discriminant analysis
RI	Retention index
LOD	Limit of detection
LOQ	Limit of quantification
RSD	Relative standard deviation
