# Supplementary material for: Molecular Sensory and GC-MS/MS Profiling of Key Aroma Compounds in Sauce-Flavor Base Baijiu
Source: Foods. 2026 Oct 17;15(20):3698. doi: 10.3390/foods15203698 (PMC13704351; doi:10.3390/foods15203698)
Supplement: Supplementary file 1 [file foods-15-03698-s001.zip › foods-4569175-supplementary.pdf]

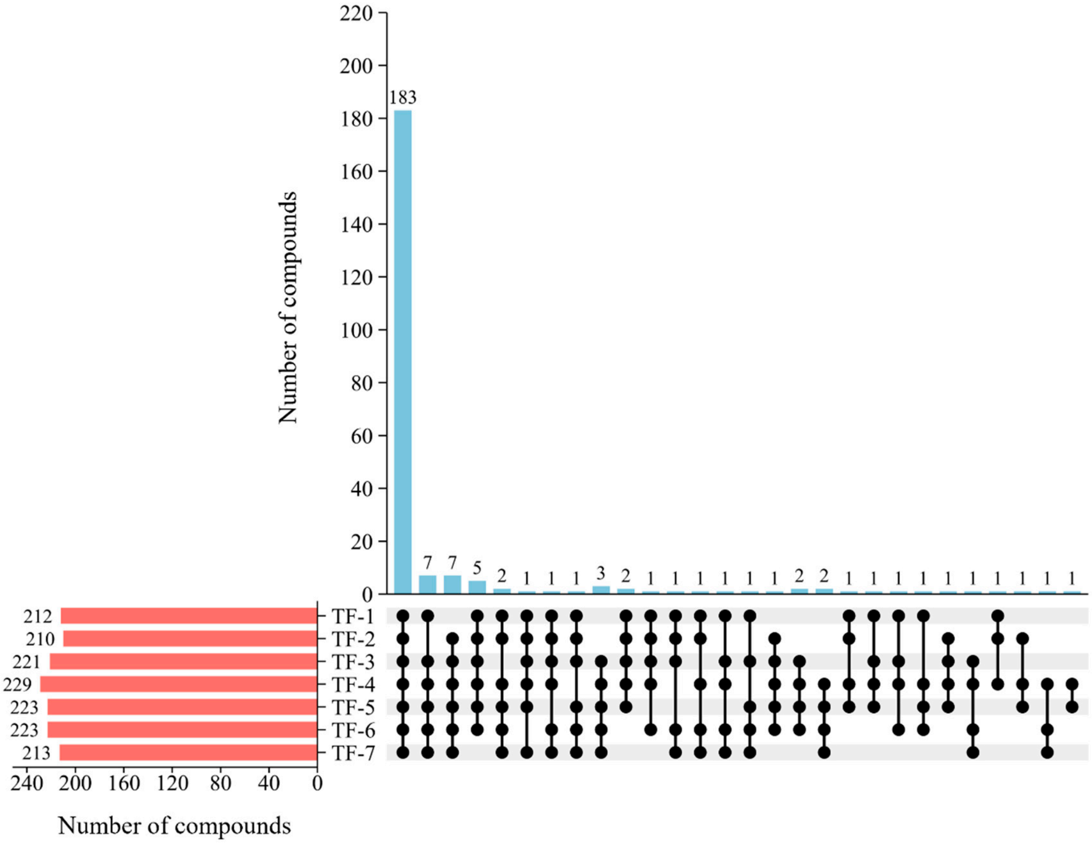

Figure S1. Distribution of volatile compounds tentatively identified by GC-MS based on NIST 2020 library matching in Sauce-flavor base baijiu across seven production rounds.

Table S1. Method validation results for high-content aroma compounds

Aroma compounds	Retention time (min)	Linear ranges (mg/L)	Calibration curves	R^2	Recovery rate (%)	RSD (%)	
						Intraday	Interday
Ethyl acetate	8.438	100-5000	y=0.5064x+0.0892	0.9971	93.38	4.09	7.88
2-Butanol	12.66	2-1000	y=0.7189x+0.0154	0.9999	102.31	4.72	6.34
1-Propanol	13.028	100-30000	y=0.7230x+0.1961	0.9972	91.69	5.95	7.24
2-Methyl-1-propanol	15.155	10-500	y=0.9545x+0.0226	0.9972	93.42	3.24	7.91
1-Butanol	17.067	4-200	y=0.9127x+0.0132	0.9945	85.56	3.17	3.15
3-Methyl-1-butanol	19.5	20-1000	y=1.1572x-0.0509	0.9962	91.71	6.41	8.93
Ethyl lactate	24.568	80-4000	y=0.5804x+0.0840	0.9994	94.17	5.81	6.33
Acetic acid	28.61	100-4000	y=0.4229x-0.2231	0.9966	103.57	7.08	6.81
Furfural	29.047	2-1000	y=0.7775x+0.0461	0.9979	97.58	3.12	3.28
Propanoic acid	31.737	4-200	y=0.6889x-0.0032	0.9963	101.02	4.87	6.62

2,3-Butanediol	33.097	4-200	y=0.5932x-0.0115	0.9961	88.27	7.42	3.16
Ethyl palmitate	53.255	10-500	y=0.9666x-0.0571	0.9964	99.42	3.32	8.84

Note: LOD and LOQ were not determined for the GC-FID method because GC-FID was used specifically for high-content aroma compounds, for which trace-level sensitivity was not an analytical objective. For the calibration equations, x represents the analyte concentration (mg/L), and y represents the analyte-to-corresponding-internal-standard peak-area ratio. Calibration standards and unknown samples were processed using the same internal-standard calculation procedure.

Table S2. Method validation results for low-content aroma compounds

Aroma compounds	Retention time (min)	Linear ranges (mg/L)	Calibration curves	R^2	LOD (μg/L)	LOQ (μg/L)	Recovery rate (%)	RSD (%)	
								Intraday	Interday
Ethyl butyrate	6.87	0.003-9	y=0.1351x+15.7430	0.9990	1.34	4.47	91.44	2.82	3.24
Propyl propionate	6.96	0.003-9	y=0.0536x+2.4778	0.9992	2.59	8.63	93.56	3.26	4.05
Ethyl isovalerate	7.23	0.003-9	y=0.0923x+6.3281	0.9990	18.47	61.58	112.56	2.07	4.35
Isoamyl acetate	7.92	0.003-9	y=0.1370x+7.2686	0.9994	35.05	116.85	96.77	3.02	3.98
Ethyl hexanoate	9.46	0.003-9	y=0.1000x+0.0373	0.9996	2.18	7.25	108.37	2.57	3.69
4-Ethoxy-2-butanone	10.28	0.003-9	y=0.0478x+2.9452	0.9994	3.04	10.13	111.71	3.33	4.02
Ethyl furfuryl ether	10.47	0.003-9	y=0.1147x+3.3802	0.9991	2.74	9.13	104.92	1.86	3.85
2-Methylpyrazine	10.57	0.003-9	y=0.0060x+0.1807	0.9994	1.49	4.95	95.49	3.58	3.75
Acetoin	11.04	0.003-9	y=0.0036x+0.6508	0.9936	66.46	221.54	111.86	3.22	4.12
2,5-Dimethyl pyrazine	11.52	0.003-9	y=0.0634x+0.5667	0.9996	1.62	5.38	90.03	3.16	4.52
2,3-Dimethyl pyrazine	12.02	0.003-9	y=0.2378x+3.1984	0.9995	0.39	1.30	104.72	3.11	3.65
2-Ethyl-6-methylpyrazine	12.7	0.003-9	y=0.3701x+3.2247	0.9998	0.58	1.92	98.54	1.51	2.64
Trimethylpyrazine	13.19	0.003-9	y=0.0857x+1.3318	0.9997	1.22	4.05	93.29	3.15	3.91
Propyl lactate	13.47	0.003-9	y=0.0232x+0.8207	0.9969	2.51	8.35	105.87	3.20	4.44
1-Octen-3-ol	13.72	0.003-9	y=0.0420x+1.9774	0.9996	1.63	5.42	106.33	2.62	3.28
3-Ethyl-2,5-dimethylpyrazine	14.05	0.003-9	y=0.3166x-6.6194	0.9999	0.76	2.54	98.86	2.60	4.67
Tetramethylpyrazine	14.86	0.003-9	y=0.1426x-1.2345	0.9999	0.59	1.96	94.42	3.45	4.04
2-Acetylfuran	15.81	0.003-9	y=0.3075x+6.0562	0.9999	0.49	1.63	104.46	2.59	4.38
Furfuryl acetate	16.32	0.003-9	y=0.1169x+1.0415	0.9999	0.26	0.85	93.82	2.32	3.88

Linalool	16.38	0.003-9	y=0.0932x+0.2429	0.9999	3.46	11.52	88.39	2.95	4.11
Ethyl 2-hydroxy-4-methylpentanoate	16.5	0.003-9	y=0.2027x-1.5144	0.9998	1.09	3.62	95.91	2.57	3.56
Isoamyl lactate	17.31	0.003-9	y=0.0522x+1.9278	0.9986	10.60	35.33	90.01	2.45	3.79
Isobutyric acid	17.37	0.003-9	y=0.0146x+1.5799	0.9909	16.03	53.44	91.99	2.23	4.21
2-Acetylpyridine	18.78	0.003-9	y=0.1005x+1.3121	0.9999	3.34	11.13	85.95	2.91	4.06
2-Acetyl-5-methylfuran	19.08	0.003-9	y=0.3583x+2.4288	0.9998	0.91	3.04	109.59	2.69	3.42
Ethyl 2-furoate	19.09	0.003-9	y=0.1296x+1.4375	0.9998	0.61	2.03	96.11	1.54	3.38
Butanoic acid	19.25	0.003-9	y=0.0298x+10.9392	0.9934	8.72	29.08	113.59	3.58	3.63
Benzeneacetaldehyde	20.05	0.003-9	y=0.0783x-1.4910	0.9995	19.18	63.92	111.81	1.78	3.51
Isovaleric acid	20.56	0.003-9	y=0.0174x+1.5597	0.9986	6.73	22.43	86.13	3.41	3.55
Diethyl succinate	20.6	0.003-9	y=0.2986x-1.0327	0.9999	0.34	1.13	96.43	1.83	2.94
(2,2-diethoxyethyl)-benzene	22.07	0.003-9	y=0.1425x-1.4866	0.9988	0.43	1.42	87.86	2.79	3.49
1-Decanol	23.26	0.003-9	y=0.0076x-0.1045	0.9996	0.89	2.97	91.33	1.81	4.74
Ethyl phenylacetate	24.47	0.003-9	y=0.0561x-0.1650	0.9977	0.52	1.74	99.67	2.76	3.24
3-Methylpentanoic acid	24.63	0.003-9	y=0.0063x-0.3978	0.9955	10.66	35.55	107.46	3.63	3.76
Phenethyl acetate	25.51	0.003-9	y=0.2105x-2.7540	0.9992	0.63	2.12	92.38	3.91	4.85
Ethyl nicotinate	25.62	0.003-9	y=0.1158x+1.1251	0.9986	1.76	5.88	99.42	2.40	3.81
Methyl cyclopentenolone	25.97	0.003-9	y=0.0293x+0.6767	0.9992	1.65	5.50	92.08	3.18	4.25
Ethyl laurate	26.03	0.003-9	y=0.1003x-2.0779	0.9986	1.76	5.88	91.71	3.34	3.59
Geraniol	26.2	0.003-9	y=0.0061x-0.1152	0.9994	2.76	9.19	90.83	3.51	4.05
Hexanoic acid	26.59	0.003-9	y=0.0087x+1.2580	0.9952	3.69	12.29	112.41	2.85	4.16
Geranylacetone	26.61	0.003-9	y=0.0085x-0.1523	0.9993	9.26	30.86	102.16	2.82	3.94
S-Methyl thiofuroate	26.74	0.003-9	y=0.0790x+0.3645	0.9986	0.26	0.87	104.27	2.59	2.56
Guaiacol	27.06	0.003-9	y=0.1013x+0.0692	0.9996	1.26	4.21	102.43	2.79	4.73
Benzyl alcohol	27.54	0.003-9	y=0.0576x-0.2214	0.9997	3.74	12.47	94.69	1.61	3.06
Ethyl 3-phenylpropionate	27.8	0.003-9	y=0.0638x+1.3842	0.9903	3.84	12.81	91.05	1.83	3.58
Phenylethyl alcohol	28.71	0.003-9	y=0.0697x+1.8509	0.9991	1.37	4.58	114.78	3.91	2.92
2-Phenyl-2-butenal	29.52	0.003-9	y=0.0330x-0.6182	0.9999	2.68	8.93	89.06	2.96	3.44
Creosol	30.22	0.003-9	y=0.0923x-1.2129	0.9987	0.69	2.30	111.97	2.61	3.56
2-Acetyl pyrrole	30.74	0.003-9	y=0.1403x+5.9377	0.9969	0.64	2.12	97.08	2.89	4.56
Phenol	31.88	0.003-9	y=0.0691x+2.4912	0.9999	5.19	17.31	110.03	3.39	3.41

4-Ethyl-2-methoxyphenol	32.59	0.003-9	y=0.1150x-3.6415	0.9995	0.72	2.40	95.21	3.20	3.47
γ-Nonalactone	32.75	0.003-9	y=0.0735x-0.3072	0.9999	3.26	10.86	101.79	3.36	4.93
3-Phenylpropanol	33.02	0.003-9	y=0.0943x-0.7384	0.9998	1.17	3.91	92.46	3.22	3.25
Octanoic acid	33.38	0.003-9	y=0.0083x-0.3042	0.9935	1.44	4.80	109.27	1.64	4.05
γ-Decalactone	36.35	0.003-9	y=0.0617x+0.2469	0.9999	0.35	1.17	85.01	1.87	3.65
Eugenol	36.9	0.003-9	y=0.0334x-0.7997	0.9997	2.80	9.34	86.12	3.21	4.83
4-Ethylphenol	37.16	0.003-9	y=0.1433x-1.2047	0.9999	1.64	5.46	113.05	2.64	3.11
2-Methoxy-4-vinylphenol	37.82	0.003-9	y=0.0580x-0.3983	0.9998	1.00	3.34	111.87	2.05	3.48

Note: The calibration ranges refer to analyte concentrations in the solutions subjected to GC-MS/MS analysis. Samples exceeding the upper calibration limit were diluted tenfold before analysis, except for propyl lactate, which was diluted twentyfold. Concentrations reported in Table S4 correspond to the original samples after correction for the respective dilution factors.

Table S3. Quantitative results of high-content aroma compounds (mg/L)

Aroma compounds	TF-1	TF-2	TF-3	TF-4	TF-5	TF-6	TF-7
Ethyl acetate	5018.87±87.59	4157.20±55.82	3018.55±46.92	1766.29±18.11	1841.86±24.84	1644.52±51.51	1592.16±19.42
2-Butanol	400.16±5.66	19.13±0.98	6.28±0.46	4.70±0.39	14.73±0.98	52.76±4.06	62.84±1.79
1-Propanol	26118.24±71.17	3289.82±34.90	726.09±18.34	404.39±2.03	933.28±3.86	2294.05±84.05	2254.90±20.55
2-Methyl-1-propanol	297.45±1.62	142.57±0.93	187.25±4.55	150.17±3.18	151.54±2.03	205.36±0.70	275.93±3.40
1-Butanol	12.12±0.30	9.54±0.52	14.20±1.44	12.73±1.09	22.89±1.27	53.31±2.72	87.42±0.86
3-Methyl-1-butanol	437.76±1.47	224.59±0.73	256.30±3.10	266.96±1.24	333.72±1.58	450.09±4.79	605.15±6.73
Ethyl lactate	1549.62±8.93	2240.17±15.00	2586.84±101.73	2838.41±111.49	2508.68±15.22	1439.91±49.97	1182.68±15.85
Acetic acid	3080.95±31.09	2665.08±11.52	2027.28±33.10	1366.29±9.41	1326.09±4.47	944.20±14.45	922.90±8.30
Furfural	6.76±0.99	43.55±2.28	120.69±6.23	245.00±0.69	322.07±3.72	300.04±6.67	346.31±4.39
Propanoic acid	190.19±8.37	51.36±1.64	43.47±2.64	44.86±0.30	45.65±2.48	40.86±3.11	42.43±1.24
2,3-Butanediol	31.45±2.22	32.33±2.21	42.81±6.67	44.81±0.25	57.09±4.39	41.21±2.53	49.21±3.60
Ethyl palmitate	61.75±0.82	61.16±1.42	55.97±2.06	66.87±1.22	70.55±2.17	64.89±1.03	67.15±1.48

Note: Concentrations correspond to the original Sauce-flavor baijiu samples. Samples exceeding the upper GC-FID calibration limit were diluted 50-fold before analysis, with the internal-standard mixture added after dilution to achieve the specified final concentration in the analyzed solution. Reported concentrations were back-calculated using the corresponding dilution factor.

Table S4. Quantitative results of low-content aroma compounds (μg/L)

Aroma compounds	TF-1	TF-2	TF-3	TF-4	TF-5	TF-6	TF-7
Ethyl butyrate	3408.31±56.54	2359.87±51.74	2238.86±68.44	2011.52±62.82	3866.70±349.07	5041.84±377.77	7320.45±453.89
Propyl	8068.29±1185.9	614.56±44.46	39.75±3.82	12.58±0.84	173.76±16.85	540.63±0.07	890.79±50.43

propionate	2						
Ethyl isovalerate	3284.42±106.87	2805.84±33.15	3181.91±58.84	2660.76±108.11	5110.77±373.20	2894.93±119.12	3248.25±192.37
Isoamyl acetate	14030.18±207.80	6114.62±176.20	5071.19±77.87	2732.78±275.76	5857.55±400.02	1546.35±117.40	7240.01±140.88
Ethyl hexanoate	1677.45±93.74	1081.27±78.46	1090.79±70.52	879.76±75.56	1095.19±31.58	801.96±63.11	824.02±21.92
4-Ethoxy-2-butanone	128.72±8.89	255.97±10.84	351.63±19.86	407.75±17.12	460.95±27.08	495.52±30.37	512.89±36.70
Ethyl furfuryl ether	1860.62±27.96	3214.04±295.43	7638.20±147.24	12158.46±1245.86	31625.29±1970.27	36793.02±1298.02	33383.84±986.95
2-Methylpyrazine	189.75±12.26	153.09±11.80	191.14±10.59	222.99±15.65	344.51±27.36	573.49±43.62	897.87±13.69
Acetoin	3372.75±139.93	12165.59±580.58	39670.03±1451.60	85239.78±278.44	36932.38±2421.92	18615.22±398.68	18029.24±600.13
2,5-Dimethyl pyrazine	138.88±11.78	107.64±3.28	100.02±6.37	117.85±10.28	169.97±5.40	299.88±8.23	449.96±21.38
2,3-Dimethyl pyrazine	395.02±9.53	305.45±7.29	269.08±13.75	274.70±21.36	353.85±10.13	357.57±17.37	352.24±9.85
2-Ethyl-6-methylpyrazine	105.36±8.73	94.70±3.78	185.31±7.33	308.86±12.99	574.46±18.83	614.13±10.44	759.59±18.91
Trimethylpyrazine	1373.18±32.81	1058.49±54.41	928.15±33.34	952.70±90.76	1412.60±41.40	1607.63±120.10	1681.42±68.00
Propyl lactate	112969.11±1954.18	31633.61±869.09	7426.06±546.03	3460.51±48.59	6196.45±268.58	8658.29±705.67	7166.34±291.88
1-Octen-3-ol	28.63±2.00	12.29±0.95	56.52±5.27	65.10±5.28	47.34±3.40	63.58±0.90	108.85±9.20
3-Ethyl-2,5-dimethylpyrazine	44.51±2.17	39.82±1.33	39.26±0.56	45.55±0.99	56.28±1.79	78.36±1.31	92.52±2.55
Tetramethylpyrazine	10732.28±293.54	8000.50±590.28	6920.53±517.98	5708.98±208.32	8175.61±294.77	7888.50±369.20	7522.46±301.17
2-Acetylfuran	287.50±1.75	714.56±14.91	1035.59±36.94	1310.18±83.14	1805.49±64.62	2245.41±97.76	2548.93±41.58
Furfuryl acetate	225.05±4.30	282.72±8.26	432.11±8.01	544.45±7.31	702.27±7.86	905.37±36.24	1145.93±30.64
Linalool	37.45±0.40	39.23±2.82	36.23±0.89	42.06±3.78	46.79±2.20	52.09±3.71	49.44±3.41
Ethyl 2-hydroxy-4-methylpentanoate	7412.02±114.18	10882.20±429.02	14999.38±517.06	11290.40±1109.68	15085.78±711.10	13517.98±200.22	9300.37±302.00
Isoamyl lactate	5464.78±88.89	3719.34±147.08	4544.94±321.81	4563.14±63.49	5760.28±189.59	6563.20±539.67	7511.02±359.58
Isobutyric acid	24564.37±792.57	23989.31±1366.56	28134.85±1667.42	29290.67±54.02	36851.41±3334.92	33679.26±561.99	41010.16±1019.00
2-Acetylpyridine	15.73±1.66	21.12±2.20	25.55±1.06	32.09±0.34	50.14±4.94	54.24±2.09	55.51±5.73
2-Acetyl-5-methylfuran	43.05±4.34	111.44±7.25	343.87±26.51	632.50±25.09	882.39±37.50	923.13±57.55	924.90±35.75

Ethyl 2-furoate	40.36±1.06	69.12±0.98	212.57±10.21	465.39±27.11	622.82±11.93	380.63±9.04	369.94±11.41
Butanoic acid	7037.96±990.69	7363.37±805.83	5579.34±641.12	6516.08±104.57	9758.87±878.19	10525.80±961.82	19875.19±403.01
Benzeneacetaldehyde	1060.25±67.18	1657.73±136.66	2617.64±157.55	4134.15±209.74	5648.35±150.29	7305.99±746.07	9247.44±257.13
Isovaleric acid	31994.18±136.46	29820.21±1328.23	34914.55±1688.26	32878.82±1321.55	44392.75±3992.43	38511.61±1478.09	46398.19±446.08
Diethyl succinate	748.36±7.19	1131.45±84.23	1239.03±114.95	1798.14±53.31	2428.09±133.04	1536.75±36.60	1341.29±73.67
(2,2-diethoxyethyl)-benzene	577.09±26.84	920.41±23.97	1548.29±33.81	2422.55±80.88	3421.44±310.42	4003.08±349.00	5061.84±296.60
1-Decanol	30.16±0.97	27.58±3.00	34.19±2.53	37.17±3.62	44.58±1.24	45.51±1.04	44.89±2.41
Ethyl phenylacetate	1944.87±130.41	2172.75±238.75	3936.50±318.79	5299.73±274.41	6942.31±491.13	7866.60±394.17	7215.88±547.04
3-Methylpentanoic acid	165.94±9.48	142.63±6.58	180.83±10.14	171.00±15.32	166.91±0.48	142.21±5.44	149.44±4.50
Phenethyl acetate	846.25±36.72	825.45±69.10	1198.90±71.56	1263.56±132.36	1822.21±109.19	3303.17±83.07	3689.22±230.68
Ethyl nicotinate	59.75±5.47	50.12±4.10	44.45±1.18	56.54±3.46	46.30±1.28	26.51±1.06	19.71±1.90
Methyl cyclopentenolone	28.00±2.84	19.01±2.07	34.90±3.59	53.38±4.25	62.97±3.45	58.35±3.36	86.49±6.26
Ethyl laurate	214.43±5.37	155.58±10.54	246.95±14.98	500.75±26.69	402.31±37.65	442.77±16.96	500.66±38.88
Geraniol	72.98±7.50	44.87±2.56	45.40±1.47	45.15±2.07	43.05±4.44	35.88±3.44	35.71±1.99
Hexanoic acid	2608.08±161.76	2004.20±159.49	2062.34±110.79	2536.28±34.31	1771.60±59.32	1548.03±74.21	2524.24±124.14
Geranylacetone	44.67±1.80	52.38±4.65	68.02±4.46	70.28±7.10	78.83±5.25	93.15±2.54	97.95±7.92
S-Methyl thiofuroate	0	0	0	0	3.12±0.26	3.53±0.29	5.31±0.18
Guaiacol	74.55±2.78	54.62±4.38	27.49±0.53	25.32±0.74	46.51±4.62	65.00±4.81	53.62±4.09
Benzyl alcohol	168.82±11.50	188.22±7.21	283.13±4.04	425.06±7.57	502.55±11.21	595.14±24.58	792.36±10.99
Ethyl 3-phenylpropionate	192.76±12.16	140.20±12.31	145.18±12.92	127.87±6.37	133.79±9.76	123.38±4.03	131.31±3.17
Phenylethyl alcohol	7235.59±336.00	8969.13±260.38	14290.35±5.81	29214.71±46.26	18394.78±238.728	16702.62±281.78	29927.35±473.38
2-Phenyl-2-butenal	23.02±1.77	40.63±1.71	53.16±1.57	57.24±2.44	60.67±1.63	80.91±3.58	105.11±8.37
Creosol	25.47±1.36	32.80±2.88	15.74±0.15	14.79±0.20	15.70±1.03	16.92±1.67	15.98±1.44
2-Acetyl pyrrole	82.29±6.33	147.28±6.41	204.63±4.18	270.33±18.55	298.27±16.68	271.37±16.47	283.12±12.13
Phenol	249.49±15.92	157.73±12.67	121.76±8.24	76.13±2.80	61.92±5.50	50.83±4.72	54.31±3.43
4-Ethyl-2-methoxyphenol	40.82±2.62	47.36±3.79	35.39±3.12	34.18±1.93	34.89±1.66	34.77±1.48	34.38±1.06

γ-Nonalactone	115.29±1.68	110.10±5.11	106.05±3.48	108.81±1.31	124.27±4.97	95.19±7.37	109.14±2.25
3-Phenylpropanol	16.31±2.84	17.79±0.62	18.84±0.25	24.24±1.37	28.48±2.17	26.50±1.68	35.44±0.26
Octanoic acid	914.66±49.71	947.34±96.51	1085.27±28.80	1173.60±95.57	1390.25±68.16	962.37±45.05	889.03±31.96
γ-Decalactone	0	0	0	6.17±0.47	5.49±0.42	3.08±0.17	4.48±0.36
Eugenol	25.99±2.24	25.39±0.40	24.90±0.30	26.88±2.35	27.24±2.88	25.13±1.33	25.13±0.89
4-Ethylphenol	137.23±14.00	105.28±9.26	68.35±5.36	43.44±2.98	36.71±1.31	26.57±2.04	26.27±1.70
2-Methoxy-4-vinylphenol	22.48±2.05	27.38±0.50	16.62±1.04	15.09±1.16	18.01±1.66	17.86±0.27	14.62±0.42

Note: Concentrations correspond to the original Sauce-flavor baijiu samples. Samples containing analytes above the upper calibration limit were diluted tenfold before GC-MS/MS analysis, except for propyl lactate, which was diluted twentyfold. The reported concentrations were calculated using the corresponding dilution factors. When dilution was required, samples were diluted before IS1 and IS2 were added to their final concentration of 1 mg/L in the solution subjected to GC-MS/MS analysis. Calibration standards and unknown samples were prepared and calculated using the same internal-standard procedure.

Table S5. Aroma omission test results for Sauce-flavor base baijiu from representative production rounds.

No.	Aroma compounds omitted from the recombinant model	Odor descriptor	n [a]		
			TF-1	TF-4	TF-7
1	Ethyl furfuryl ether	sweet, spicy	8	7	9
2	Ethyl isovalerate	apple, pineapple	9	8	8
3	1-Propanol	pungent, bitter	7	3	5
4	Acetoin	butter, creamy	3	8	7
5	Ethyl acetate	fruity, with baijiu aroma	9	6	5
6	Isoamyl acetate	apple, pear	7	4	4
7	Ethyl butyrate	pineapple, fruity	3	4	3
8	Ethyl hexanoate	apple peel, brandy	2	4	3
9	Isovaleric acid	sour odor, cheese	4	9	4
10	Isobutyric acid	cheese, sweat	5	5	4
11	Ethyl lactate	fruity	6	4	2
12	Acetic acid	acid, vinegar	8	6	3
13	2-Methyl-1-propanol	apple, bitter	4	8	3
14	Butanoic acid	sour odor, cheese	4	4	3
15	1-Octen-3-ol	mushroom	6	3	7
16	3-Ethyl-2,5-dimethylpyrazine	roasted	4	5	3
17	Ethyl phenylacetate	rose, honey	5	6	5
18	1-Butanol	fruity	3	5	5
19	Benzeneacetaldehyde	floral, apple	4	5	3
20	Propanoic acid	sour odor, sweat	5	5	3
21	2-Ethyl-6-methylpyrazine	nutty, roasted	5	6	2
22	Ethyl 2-hydroxy-4-methylpentanoate	blackberry, fruity	8	4	4

23	Guaiacol	burnt, wood	5	5	8
24	Propyl propionate	apple, banana	6	—	3
25	2-Butanol	wine	2	—	2
26	3-Methyl-1-butanol	burnt, malty	3	4	8
27	Trimethylpyrazine	potato, roasted	6	5	2
28	Ethyl 3-phenylpropionate	fruity, rose	6	4	2
29	Ethyl palmitate	oil	5	4	4
30	Linalool	ether, lemon	3	2	4
31	Phenethyl acetate	floral, honey	5	5	3
32	Hexanoic acid	cheese, Daqu	4	5	3
33	γ -Nonalactone	coconut	4	6	2
34	Eugenol	clove	5	6	5
35	Furfural	almond, baked potatoes	—	6	5
36	Ethyl laurate	fruity, fat odor	—	4	5
37	Phenylethyl alcohol	honey, clove	—	5	2

Note: [a] Number of correct responses from ten assessors in the triangle test. Under the null hypothesis, the probability of a correct response by chance was 1/3. Seven, eight, and ⩾nine correct responses corresponded to $p < 0.05$, $p < 0.01$, and $p < 0.001$, respectively. — indicates that the compound was not included in, and therefore was not tested for, the corresponding recombination model.

Table S6. Composition and final concentrations of aroma compounds included in the recombination models of TF-1, TF-4, and TF-7.

No.	Aroma compound	Final concentration in recombination model (mg/L)		
		TF-1	TF-4	TF-7
1	Ethyl furfuryl ether	1.86	12.16	33.38
2	Ethyl isovalerate	3.28	2.66	3.25
3	1-Propanol	26118.24	404.39	2254.90
4	Acetoin	3.37	85.24	18.03
5	Ethyl acetate	5018.87	1766.29	1592.16
6	Isoamyl acetate	14.03	2.73	7.24
7	Ethyl butyrate	3.41	2.01	7.32
8	Ethyl hexanoate	1.68	0.88	0.82
9	Isovaleric acid	31.99	32.88	46.40
10	Isobutyric acid	24.56	29.29	41.01
11	Ethyl lactate	1549.62	2838.41	1182.68
12	Acetic acid	3080.95	1366.29	922.90
13	2-Methyl-1-propanol	297.45	150.17	275.93
14	Butanoic acid	7.04	6.52	19.88
15	1-Octen-3-ol	0.03	0.07	0.11
16	3-Ethyl-2,5-dimethylpyrazine	0.04	0.05	0.09
17	Ethyl phenylacetate	1.94	5.30	7.22
18	1-Butanol	12.12	12.73	87.42
19	Benzeneacetaldehyde	1.06	4.13	9.25

No.	Aroma compound	Final concentration in recombination model (mg/L)		
		TF-1	TF-4	TF-7
20	Propanoic acid	190.19	44.86	42.43
21	2-Ethyl-6-methylpyrazine	0.11	0.31	0.76
22	Ethyl 2-hydroxy-4-methylpentanoate	7.41	11.29	9.30
23	Guaiacol	0.07	0.03	0.05
24	Propyl propionate	8.07	—	0.89
25	2-Butanol	400.16	—	62.84
26	3-Methyl-1-butanol	437.76	266.96	605.15
27	Trimethylpyrazine	1.37	0.95	1.68
28	Ethyl 3-phenylpropionate	0.19	0.13	0.13
29	Ethyl palmitate	61.75	66.87	67.15
30	Linalool	0.04	0.04	0.05
31	Phenethyl acetate	0.85	1.26	3.69
32	Hexanoic acid	2.61	2.54	2.52
33	γ-Nonalactone	0.12	0.11	0.11
34	Eugenol	0.03	0.03	0.03
35	Furfural	—	245.00	346.31
36	Ethyl laurate	—	0.50	0.50
37	Phenylethyl alcohol	—	29.21	29.93

Note: Values represent the mean quantitative concentrations used as final target concentrations in the corresponding recombination mixtures. Values for high-content compounds were obtained directly from Table S3. For low-content compounds reported in Table S4 in μg/L, the corresponding values were converted to mg/L. "—" indicates that the compound was not included in the corresponding recombination model because its OAV was < 1. The TF-1, TF-4, and TF-7 recombination models contained 34, 35, and 37 compounds, respectively. The hydroalcoholic matrices contained 56.6%, 53.5%, and 52.5% ethanol (v/v) for TF-1, TF-4, and TF-7, respectively.

Table S7. Raw sensory intensity scores assigned by ten trained assessors to 12 aroma attributes of Sauce-flavor base baijiu across seven production rounds.

Assessor	Sample	Sauce	Raw grain	Ripe grain	Raw qu	Aged qu	Floral	Fruity	Sweet	Sour	Green	Zao	Baked
A01	TF-1	4	3	4	2	3	3	4	3	4	3	0	2
A01	TF-2	3	4	4	2	4	3	0	1	3	4	0	4
A01	TF-3	4	0	5	3	4	2	1	3	4	3	1	2
A01	TF-4	5	0	5	1	5	2	4	4	4	3	1	2
A01	TF-5	5	0	4	0	5	0	0	0	3	4	0	1
A01	TF-6	5	0	4	1	4	0	1	1	2	0	3	0
A01	TF-7	5	1	4	0	4	0	0	0	4	1	1	3
A02	TF-1	3	2	4	2	3	2	3	4	3	3	0	2
A02	TF-2	3	5	4	2	4	2	0	2	3	3	1	3

A02	TF-3	5	0	5	2	4	2	1	4	4	4	2	3
A02	TF-4	4	1	5	0	5	2	3	3	4	3	2	2
A02	TF-5	5	0	4	0	4	0	0	1	4	4	0	0
A02	TF-6	5	0	4	0	4	0	0	0	4	0	3	1
A02	TF-7	5	0	2	1	4	0	0	1	3	0	1	3
A03	TF-1	3	2	4	3	4	4	3	2	4	4	0	2
A03	TF-2	3	4	3	2	4	2	0	2	3	2	1	3
A03	TF-3	4	0	5	2	4	2	1	2	3	3	2	3
A03	TF-4	5	1	5	1	5	2	4	3	5	3	1	2
A03	TF-5	5	0	4	0	5	1	0	0	2	2	0	0
A03	TF-6	5	0	2	1	4	1	0	0	4	1	4	0
A03	TF-7	5	0	3	0	4	0	0	0	3	0	0	3
A04	TF-1	4	2	4	2	3	3	4	2	4	2	0	2
A04	TF-2	2	4	4	2	3	2	1	1	2	2	0	3
A04	TF-3	4	0	4	1	4	3	1	3	5	3	0	2
A04	TF-4	5	0	5	0	5	2	4	3	3	3	1	2
A04	TF-5	5	0	4	0	5	0	0	0	3	3	1	0
A04	TF-6	5	0	3	0	4	0	0	1	3	1	3	0
A04	TF-7	5	1	4	0	4	0	0	0	3	0	0	3
A05	TF-1	2	2	3	2	2	3	3	4	4	3	0	2
A05	TF-2	3	4	4	2	4	1	0	0	2	4	1	3
A05	TF-3	4	0	5	2	4	2	1	3	4	3	1	1
A05	TF-4	5	0	4	0	5	2	3	3	4	3	2	1
A05	TF-5	4	0	4	0	5	0	0	0	2	2	0	0
A05	TF-6	4	1	4	0	5	0	0	0	3	0	3	0
A05	TF-7	4	0	3	0	4	0	0	0	3	1	0	2
A06	TF-1	3	2	4	2	4	3	3	3	5	3	1	2
A06	TF-2	4	4	5	3	5	2	0	1	3	3	2	3
A06	TF-3	4	1	5	3	5	3	2	3	5	3	1	3
A06	TF-4	5	0	5	0	5	3	5	3	4	3	1	2
A06	TF-5	5	1	4	1	5	0	1	0	2	4	0	1
A06	TF-6	5	0	3	0	5	0	0	0	3	0	3	0
A06	TF-7	5	1	3	1	5	1	1	1	4	0	0	4
A07	TF-1	4	2	4	2	2	3	3	4	4	3	1	3
A07	TF-2	3	4	4	2	5	3	1	1	3	3	1	3
A07	TF-3	4	0	5	2	4	2	1	3	5	3	1	3
A07	TF-4	5	0	5	0	5	2	4	3	4	3	1	2
A07	TF-5	5	1	4	0	5	0	0	1	4	3	0	0
A07	TF-6	4	0	3	0	4	0	1	1	3	0	3	0
A07	TF-7	5	0	3	0	4	1	0	1	3	0	0	3
A08	TF-1	2	1	4	2	4	3	2	3	4	3	0	1
A08	TF-2	3	4	3	2	4	2	0	1	3	3	1	2
A08	TF-3	4	0	4	2	4	1	1	3	4	3	0	1
A08	TF-4	4	0	5	0	4	2	4	2	4	3	0	2

A08	TF-5	5	0	3	0	5	0	0	0	4	3	0	0
A08	TF-6	5	0	3	0	3	1	0	0	3	0	2	0
A08	TF-7	5	0	3	0	4	0	0	0	3	0	0	3
A09	TF-1	3	2	3	2	3	3	4	2	4	3	0	1
A09	TF-2	3	5	3	2	4	2	0	1	3	3	2	3
A09	TF-3	5	0	5	2	3	2	1	3	4	3	1	1
A09	TF-4	5	0	5	0	5	2	4	3	4	3	1	2
A09	TF-5	5	0	5	0	5	0	0	0	3	3	0	0
A09	TF-6	5	0	3	0	4	0	0	0	3	0	3	0
A09	TF-7	5	0	3	0	4	0	0	0	3	0	0	3
A10	TF-1	2	2	4	1	2	3	3	3	4	3	0	3
A10	TF-2	3	3	4	1	3	1	0	0	3	3	0	3
A10	TF-3	4	0	4	1	4	2	1	3	4	3	0	1
A10	TF-4	4	0	4	0	4	1	4	2	4	2	1	2
A10	TF-5	5	0	4	0	4	0	0	0	2	2	0	0
A10	TF-6	5	0	2	1	3	0	0	0	3	0	3	0
A10	TF-7	4	0	3	0	4	0	0	0	3	0	0	3

Note: Values represent the individual sensory intensity scores assigned independently by ten trained assessors using a six-point intensity scale from 0 to 5, where 0 indicates the absence of the corresponding aroma attribute and 5 indicates a very strong intensity. These raw assessor-level data were used for the Friedman tests, Kendall's W calculations, and paired Wilcoxon signed-rank comparisons.
